# Supplementary material for: ‘People have started to deliver in the facility these days’: a qualitative exploration of factors affecting facility delivery in Ethiopia
Source: BMJ Open. 2019 Jun 12;9(6):e025516. doi: 10.1136/bmjopen-2018-025516 (PMC6576119; doi:10.1136/bmjopen-2018-025516)
Supplement: Author's manuscript [file bmjopen-2018-025516.draft_revisions.pdf]

BMJ Open is committed to open peer review. As part of this commitment we make the peer review history of every article we publish publicly available.

When an article is published we post the peer reviewers' comments and the authors' responses online. We also post the versions of the paper that were used during peer review. These are the versions that the peer review comments apply to.

The versions of the paper that follow are the versions that were submitted during the peer review process. They are not the versions of record or the final published versions. They should not be cited or distributed as the published version of this manuscript.

BMJ Open is an open access journal and the full, final, typeset and author-corrected version of record of the manuscript is available on our site with no access controls, subscription charges or pay-per-view fees (http://bmjopen.bmj.com).

If you have any questions on BMJ Open's open peer review process please email info.bmjopen@bmj.com

BMJ Open

"People have started to deliver in the facility these days": A qualitative exploration of factors affecting facility delivery in Ethiopia

Journal:	*BMJ Open*
Manuscript ID	bmjopen-2018-025516
Article Type:	Research
Date Submitted by the Author:	20-Jul-2018

Complete List of Authors:	Hill, Z; University College London, Institute for Global Health<br>Amare, Yared; Consultancy for Social Development<br>Scheelbeek, Pauline; University College London, Institute For Global Health<br>Schellenberg, Joanna; London School of Hygiene & Tropical Medicine, Dept of Infectious Disease Epidemiology

Keywords:	facility delivery, qualitative, ethiopia, africa, community health workers

SCHOLARONE™
Manuscripts

"*People have started to deliver in the facility these days*": A qualitative exploration of factors affecting facility delivery in Ethiopia

Dr Zelee Hill (corresponding author), University College London, 30 Guilford Street, London WC1N 1EH, UK. zelee.hill@ucl.ac.uk

Dr Yared Amare, Consultancy for Social Development, PO Box 70196, Addis Ababa, Ethiopia. yaredamare@yahoo.com

Dr Pauline Scheelbeek, University College London, 30 Guilford Street, London WC1N 1EH, UK. pauline.scheelbeek@gmail.com

Professor Joanna Schellenberg, London School of Hygiene and Tropical Medicine, Keppel Street, London, WC1E 7HT, UK. joanna.schellenberg@lshtm.ac.uk

For peer review only

Abstract

Objectives: To understand the recent rise in facility deliveries in Ethiopia
Design: A qualitative study
Setting: Four rural communities in two regions of Ethiopia
Participants: 12 narrative, 12 in-depth interviews and four focus group discussions with recently delivered women; and four focus group discussions with each of grandmothers, fathers and community health workers.
Results: We found that several interwoven factors led to the increase in facility deliveries, and that the importance of these factors varied over time. The initial catalysts were a saturation of messages around facility delivery, improved accessibility of facilities, the prohibition of TBAs, and elders having less influence on deciding the place of delivery. Once people started to deliver in facilities, the drivers of the behavior changed. As more women began delivering in facilities, families shared positive experiences of the facilities, leading to others deciding to deliver in a facility.
Conclusion: Our findings highlight the need to employ strategies that act at multiple levels, and that both push and pull families to health facilities.

Key words: Facility delivery; Ethiopia, Qualitative

Word count: 4355

Article Summary: Strengths and limitations:

- The use of qualitative methods to understand a complex issue
- Use of multiple study sites, purposive sampling to saturation, reflexivity, triangulation of methods and respondent groups, and within and cross case analysis
- The study sites were all reasonably accessible and had reasonably functioning HEW systems. Accessibility issues may be more important in other areas of Ethiopia.
- There is the potential for social desirability bias given the pressure for women to deliver in a health facility

Funding statement: This work was supported by the Bill and Melinda Gates Foundation as part of the IDEAS study

Competing interests: The authors have no competing interests

Data sharing statement: Unpublished data from this study are not available. Data anonymity is not obtainable given the depth of the narrative qualitative transcripts, even when these have been anonymised.

For peer review only

Introduction

Sub-Saharan Africa accounts for an estimated 66% of all maternal deaths [1]. Despite recent declines, mortality rates are still unacceptably high. Most deaths are preventable and occur around the time of delivery. Although it is essential to address the broad determinants of maternal mortality, such as female education and social status [2], having a skilled attendant at delivery is still considered to be the most critical intervention [3 4].

Ethiopia had one of the lowest coverage levels of facility deliveries in the world, with Demographic and Health Survey (DHS) data showing rates of 5% in 2000, 6% in 2005, and 11% in 2011 (three year recall) [5]. This low coverage persisted despite government efforts to increase the accessibility and availability of services [6 7]. Efforts included the introduction of the Health Extension Program in 2003, where two female Health Extension Workers (HEW), stationed at newly constructed health posts, and paid around $100 a month, were trained to serve around 5,000 people. They provide health promotion, disease prevention and treatment, and work both at the health post and in the community [8-11]. The program had little impact on the coverage of skilled delivery, even when Health Extension Workers were trained to conduct deliveries in the health post [7 12], and studies have found persistent and multiple barriers to change [13-20].

By 2016 the DHS show coverage increased to 33% (3 year recall, with a regional range of 18-97%), a 200% increase from 2011 [21]. The Ethiopian government attributes the increase largely to the work of the Health Development Army (HDA) [22-24]. The HDA, created in 2012, is a network of all women in rural areas, organized into development groups of 30 women (1-30 networks), who are further clustered into groups of 6 (1-5 networks) [20 25-27]. Groups select a leader who is then trained and supervised by the HEW. The HDA leaders help members adopt practices promoted by the HEW, hold participatory learning and action meetings, link pregnant women with care providers, hold monthly meetings for pregnant women, mobilize communities to contribute resources to make facilities mother friendly, and ensure the use of either traditional or modern ambulances [11 20 22 26].

Around the same time as the creation of the HDA, there were other policy changes that could have influenced facility delivery rates. These include the prohibition of the use of Traditional Birth Attendants (TBAs) for delivery [20 25], and the provision of a four-wheel drive ambulance to transport women to facilities for delivery to every rural district [28]. In addition the number of health centers, staffed with two midwives, increased and the road infrastructure was also improved [25 29]. In this paper we use qualitative data to explore the reasons for the increase in facility deliveries in four study sites, we used a phenomenological approach as were interested in understanding lived experiences.

For peer review only

Methods:

Study setting selection and characteristics: Data were collected between March and May 2015, from two *Kebeles* in the Southern Nations, Nationalities and Peoples region (SNNP) and two in Amhara region. Amhara has shown an increase in facility deliveries from 11% (2011) to 35% (2016), and SNNP region from 7% to 33% [5].

Data were collected from areas where 'The Last Ten Kilometers' (L10K) programme was active in supporting the Health Extension Program [30-33]. *Kebeles* were selected from a list, provided by L10K, of *kebeles* considered to have a reasonably functioning HEW system. Other selection criteria were that the *kebeles* were seen as typical of the woreda, that is with no unusual characteristics, and were feasible to access by the data collection team. We have labeled these *kebele* "A-D" to maintain anonymity. Table 1 shows the characteristics of the selected *kebele*s, all of which had a subsistence farming based economy.

Table 1. Characteristics of Study *kebele*

**Region**	***kebele***	**Ethnicity**	**Predominant religion**	**Access to health centers**	**Terrain**
Amhara	*kebele* A	Amhara	Orthodox Christian	Moderate	Hilly
	kebele B	Amhara	Orthodox Christian	High	Hilly
SNNPR	*kebele* C	Gamo/ Wolaita	Protestant/ Orthodox Christian	High	Predominantly flat with some hilly parts
	kebele D	Silte	Muslim	Moderate	Flat

Data collection: Data were collected as part of a larger study to understand how HEWs influence maternal and newborn care behaviors. Four trained interviewers collected data in the local language using pre-tested semi structured guides developed by the authors. When needed translators were used. The content of the guides was informed by a theoretical framework, which identified pathways through which HEWs could influence behaviors by modifying families capabilities, opportunities and motivation [34]. Data were collected from recent mothers, grandmothers, fathers, HEWs and HDA using narrative interviews, in-depth interviews, and focus group discussions. Using a range of both methods and respondents allowed for data triangulation and ensured we captured a range of viewpoints. Data were collected until saturation was reached, i.e. additional interviews provided similar information to that already obtained. Saturation was determined by frequent transcript reviews. The sample size, respondent groups, and the interview content related to facility delivery are shown in Table 2. In the FGD we employed several activity oriented exercises such as sorting and ranking and root cause analysis to encourage group interaction and participation and reduce social desirability bias, which can be a particular issue in Ethiopia [35].

For peer review only - http://bmjopen.bmj.com/site/about/guidelines.xhtml

Table 2: Data collection method, sample size and content related to facility delivery

Method	Sample	Interview content related to facility delivery
Narrative interviews with recent mothers	12	• Labor and delivery story<br>• Perceived knowledge and skills related to pregnancy and newborn care<br>• Description of contacts with health workers, HDA and HEW<br>• Information received on where to deliver, reaction to the information, and decision making
In-depth interviews with recent mothers	13	• Perceptions of where most people deliver; community views of those who deliver at home/facility<br>• HEW/HDA roles and suitability<br>• Description of contacts with health workers, HDA and HEW<br>• Information received on where to deliver and reaction to the information<br>• Most significant maternal and newborn health changes in last 2 years, and why the change
FGD with recent mothers	4	• Pile sort of behaviors practiced/not practiced, important/not important, promoted/not promoted by HEW/HDA<br>• Community views of those who deliver at home/ facility<br>• Most significant maternal and newborn health changes in the last 2 years, and why the change<br>• Reaction to statements that HEW/HDA work does not bring change, and that people dislike HEWs telling them where to deliver
FGD with grand-mothers	4	• Reaction to a picture of a facility delivery<br>• Most significant changes in the community related to maternal and newborn health in the last 2 years and why the change<br>• Reaction to statements about grandmothers supporting traditional practices, and that mothers do not listen to grandmother advice
FGDs with fathers	4	• Reaction to a picture of a facility delivery<br>• Role in deciding place of delivery<br>• Response to a scenario where a family does not follow HEW advice<br>• Views on HEW/HDA work and their suitability<br>• Most significant changes in the community related to maternal and newborn health in the last 2 years and why the change<br>• Reaction to statements that mothers/fathers make decisions about delivery, and that people dislike HEWs telling them where to deliver
FGD with HEW and HDA	4	• Pile sort of behaviors practiced/not practiced, important/not important, promoted/not promoted by HEW/HDA<br>• Most significant changes in the community, and in their work, related to maternal and newborn health in the last 2 years and why the change<br>• Successes and challenges in encouraging behavior change<br>• Reaction to statements that mothers get punished if they do not follow advice, and that mothers prefer advice of family members

For peer review only - http://bmjopen.bmj.com/site/about/guidelines.xhtml

Mothers, grandmothers and fathers were identified either by the HEW/HDA, or through snowball sampling. Eligibility criteria were that the family had received at least one visit by an HEW or HDA. Mothers were selected to ensure diversity in age, educational level, parity, sex of newborn and socio-economic status. We also aimed to get diversity in place of delivery, but located few women who admitted they delivered at home. All of the HDAs in the study *kebeles* were invited for the HDA FGDs. As there were only 2 HEWs per *kebele* HEW FGDs included HEWs from neighboring *kebeles.* Interviewers approached potential respondents in their home, or at the health post. Three respondents refused, as they were too busy. Interviews lasted from 1 to 2 hours and took place in respondents' houses, or the health post for the HEW. FGDs were conducted with 3-7 *respondents* in neutral locations and lasted from 1.5 to 2.5 hours. HEWs and HDAs were not present during any of the interviews or FGDs with community members.

Interviews and FGDs were audio-recorded and fully transcribed by the data collectors in English as soon as possible. Data collectors met regularly during fieldwork to discuss emerging themes and to receive feedback from the senior researchers. Ethical approval was granted by the Ministry of Science and Technology in Ethiopia and the London School of Hygiene and Tropical Medicine, and written informed consent was obtained from all respondents.

Respondent and public involvement: Respondents were not directly involved in the design of the study, however the interview guides were iterative and were modified as the research progressed based reported experiences and perceptions. Some respondents were recruited through snowball sampling, i.e. where respondents suggested others they knew that were eligible for interview.

Data analysis: Analysis began during data collection through regular team meetings and reflection. A formal analysis session was held with the data collectors in the middle and at the end of data collection, this included discussion how our characteristics could have influenced how data were collected and interpreted. Once data were collected all transcripts were read several times to ensure familiarity with the data, to begin to identify notable constructs and to see the data as a whole. A deductive coding template was developed in Nvivo based on the theoretical framework that guided the interview content. Interviews and focus groups were then coded inductively within these broad themes. Coding was done by identifying the underlying meaning of each section of text and how it was different or similar to others section. Codes that contained similar concepts were then put into larger themes. Themes and codes were modified by looking for patterns, links and contradictions within themes. Data credibility was checked by triangulating data between respondent groups and between data collection methods. Data analysis was done by three of the senior researchers, who discussed their coding regularly to enhance conceptual thinking and to increase coding rigor. Reflective notes were kept throughout the process.

For peer review only - http://bmjopen.bmj.com/site/about/guidelines.xhtml

Results

All respondent groups reported that the increase in facility delivery was recent, and that previous attempts to encourage facility delivery had limited success: '*People have started to deliver in the facility these days……. they used to give us lots of excuses like, let the cattle return back home, let the sun start setting, and let's wait for this and that; believing that the mother would deliver in the meantime….so that used to be very problematic'* [HEW FGD, *Kebele* A Amhara]. At the time of data collection, delivering in a health center was reported as the usual practice in all communities, with respondents reporting that *'everyone knows what to do'… 'no one delivers at home'*.

We found the factors that influenced facility delivery changed over time and consisted of push and pull factors. The initial uptake of facility delivery driven by a saturation of messages around facility delivery, improved accessibility of facilities, the prohibition of TBAs, and elders having less influence on deciding the place of delivery. Once people started to deliver in facilities, the drivers of the behavior changed. Women shared positive experiences of the facilities and families were influenced by what others had done.

Saturation of messages and contacts: All women interviewed reported receiving information on the importance of facility delivery, and all respondent groups knew that it was being strongly promoted at *'every opportunity'.* In the majority of cases women had received information from at least two sources, and at several time points: *'*[HDA] *tells me about it repeatedly and forcefully'* [Mother IDI, *Kebele* D SNNPR]. Information was mainly given at the health post, at home, during ANC, and at community meetings. The main sources of information were the HEWs and HDAs. The resultant high awareness levels was reported as a reason for the increase in facility delivery rates:

> *I: Why didn't you go* [to the facility] *at that time* [for previous deliveries]*?*
> *R: Because there was no one who educate you like this at that time. Nobody advise us to deliver in the health center…… My knowledge was not as such strong at that time* [Mother IDI, *Kebele* D SNNPR]

Not only did families receive information often, they received it from a source they reportedly trusted, with the HEWs reported as being knowledgeable and trustworthy because of their training and status: *'They are better than us; they teach us what they have learned. She went there* [training] *so that she could bring us some good education, we don't believe she teach us harmful advice'* [Mother FGD, *Kebele* D SNNPR]. HDA were less trusted and were viewed as people who transfer messages rather than being knowledgeable in their own right. But, they played a key role in ensuring the penetration of messages, and by informing HEWs about pregnant women: *'The leaders of this group* [1-5 group] *follow how many of them are pregnant …. The leaders know everything about their group…… And when labor starts, the leader will inform the HEWs'* [Mother IDI, *Kebele* B Amhara].

Improved accessibility: Knowledge of the ambulance service was universal. In some sites families were given the ambulance number during pregnancy. In other sites families called the HEW at the start of labor, and the HEWs then called the ambulance. The presence of the ambulance was reported as facilitating facility deliveries:

> *'If ambulance service is not started functioning in the kebele, the mother surely give birth at home'* [Mother FGD, *Kebele* A Amhara]

For peer review only - http://bmjopen.bmj.com/site/about/guidelines.xhtml

> *'This time there is no one who delivered at home, it was in our fathers time, now there is ambulance which take the mother to the health center so all women deliver there'* [Mother FGD, *Kebele* A Amhara]

Despite the reported importance of ambulances, around half of the families interviewed described a problem accessing the service. This was most often that the ambulance was busy, could not come because of heavy rain, had no fuel, or took too long to come: *'I asked* [HEW] *to call an ambulance, but there was no ambulance so we were told to use public transport'* [Grandmother FGD, *Kebele* B Amhara].

The four study sites were reasonably accessible and respondents talked of villages where health facility delivery was still very difficult because of accessibility issues: '*Not all villages are accessible, those who live in X village, they are not able to deliver in health facility. But those who live in nearest village….. It is a must to deliver in health facility since they are close'* [Mother IDI, *Kebele* A Amhara].

With the exception of fathers, only a few respondents reported that the increase in the number of health centers, free delivery care or the construction of roads had impacted on facility delivery uptake *'Formerly people think there is a payment for delivery like they pay for treatment but there is no such things … there were a lot of people who deliver at home thinking it needs money… even if she* [mother] *asked to go the husband didn't want, thinking he will be asked for money'* [Father FGD, *Kebele* D SNNPR].

Prohibition of TBAs: In all sites the use of traditional birth attendants was reported as forbidden, with a threat of sanctions for those who conduct or have a home delivery.

> *During pregnancy, they told me not to deliver at home. They said "if you deliver at home, you will be punished"….. 500 birr* [$22] *if I deliver at home* [Mother IDI, *Kebele* C SNNPR]

> *….birth attendants are not willing to assist due to fear of punishment….. the women herself will pay 2000 birr* [$88] *and the birth attendant will pay 1000 birr* [$44] [Mother IDI, *Kebele* B Amhara]

Decisions about sanctions were made at community level through the 1-5 or 1-30 networks: *'The community decided a 'Sera'* [customary law] *that if a mother delivers at home she will be fined 500 birr'* [Narrative interview, *Kebele* B Amhara]. The reported sanctions were varied, with the fines ranging from 200-2000 birr [$9-88].

Respondents reported that the possibility of sanctions was an initial reason that families started delivering in facilities:

> Interviewer (I): *What do you think brought this change*?
> Respondent (R):…. *the fear of the punishment, I don't think most of the community understood the benefit of delivering in the facility…. didn't give due attention to the lessons….. it is after we are told we will be punished* [Mother IDI, *Kebele* C SNNPR]

> *R2: There is 1 to 5, and one watch over the other, and there is also punishment; if the mother deliver at home she will be fined with 500birr.*
> *R3: There is a law they are fined …. after that people start saying hurry up please she is going to deliver* [laughing] [Father FGD, *Kebele* C SNNPR]

For peer review only - http://bmjopen.bmj.com/site/about/guidelines.xhtml

Respondents, including former TBAs, felt that the sanctions were justified and beneficial. HEWs were aware of them, and at times encouraged their use:

> *R: When a mother delivers at home and if the baby dies….the government will prosecute her for that….. We use such threats.*
> *I: Who tells them such threats?*
> *R: We call the HDA leaders [1 to 30] and then tell them that such threats may work, and then they go and tell the mothers* [HEW FGD, *Kebele* C SNNPR]

In only one site were there reports that sanctions had been used in practice, and the respondents that we interviewed who had delivered at home reported that they had been excused the sanctions: *'*[HEW said] *If you were another person I will take you to jail but you face a lot of problem that's why I left you'* [IDI mother, *Kebele* D SNNPR]. Although the respondents who delivered at home report that sanctions was not applied, they did report that the HEWs were angry with them and, in a few cases, denied them services: *'She* [HEW] *suspected that I hid and delivered at home….. and was very angry. Because she was angry then, she did not tell me things…..there was no mention of how I should be bathing the baby and the like'* [Narrative woman, *Kebele* B Amhara].

Power shift: Grandmothers had little influence on place of delivery, with respondents in all groups using words like *'we are in a different time'* and *'time has changed'.* This lack of influence was attributed to mothers being modern because of the education given by the HEW, were now more knowledgeable than their elders, and were consequently able to challenge their advice. Grandmothers were not resistant to the change in delivery location: *Today's mothers are young and modern. They easily accept new ideas……. they wouldn't like to do the traditional practice ….. since they have received the new education* [Narrative mother, *Kebele* C SNNPR]

Husbands were viewed as having the ultimate decision making power in the household and generally supported facility delivery. This support from husbands put mothers in a stronger position if they faced opposition from their elders. Fathers reported that they would *'not allow'* their wives to deliver at home, even if this was their wives preference.

Positive experiences: Respondents reported positive experience of facility delivery '*I felt very happy* [to deliver in the facility]*…. The doctors give morale, they said take it easy, be strong and the like'* [Mother IDI, *Kebele* D SNNPR]. Respondents reported that over time this positive experience reduced the importance of push factors, with pull factors gaining in importance:

> *But now, it is not that they are afraid of the punishment, they have started saying that they are going because they want to get care from the health professionals…. They have started saying that the physicians do all they can and help them deliver* [HDA FGD, *Kebele* D SNNPR]

> *Formerly the fear the penalty, but now those who delivered there* [at health center] *talk about good thing of delivering there* [Mother IDI, *Kebele* C SNNPR]

All respondents in all groups reported that facility delivery was safer and reduced deaths. The provision of an injection to stop bleeding was the most frequently mentioned benefit along with getting a vaccination for the baby, removal of dirt from the abdomen/stomach, the baby being cared for and not left alone, the facility being hygienic, delivery being less

painful and more predictable, and that the facility could deal with problems such as the baby being in the wrong position, the placenta getting stuck, or the baby being born weak:

> *I know that I will not be hurt if I deliver in the health center….. they will inject me something which stopped the excessive bleeding; I know that they will assist me if I will have any complications* [Mother IDI, *Kebele* C SNNPR]

> *Here* [home] *there is only suffering until the delivery nothing else, and we are delivering in the facility in a very relaxed way…… they measure and tell us how much time is left……but here we don't know anything, we are just laboring and wait until we deliver or die* [Mother IDI, *Kebele* D SNNPR]

All respondent groups reported that at the facility mothers receive food, drink and sometimes a cloth for the baby: *'Everything is perfect, even porridge and gruel is prepared and served to mothers in the health center. It is really good…….. Even the person who accompanied them is invited'* [Grandmother FGD, *Kebele* B Amhara]. Allowing family members into the delivery room was also received positively, especially by grandmothers as it allayed fears about what occurs during a facility delivery: *'We never used to allow anybody inside the delivery … as they start to see; they started saying 'we were afraid that you would insert materials inside her' …. they see that things are good'* [HEW FGD, *Kebele* A Amhara].

Not only did respondents report good experiences of delivering at the facility, they often shared their experience with their friends and neighbors, and several mothers reported that their friends and neighbors had influenced their delivery location:

> *I heard from other people, I heard that it is good to deliver in the health center, so I was planning to deliver there…. one of my neighbors delivered in the health facility, and she told me it is good…. She told me the mother will be very clean, she will not have bleeding….. health professionals would help the mother and saved her life*. [Mother narrative, *Kebele* C SNNPR]

> *R1: Now, the unwilling ones also went there because she saw when others do.*
> *R2: Yes, formerly, they did not want to be exposed. They said that, Saint Mary will do what she wants. But now, they see the benefit. And learn one from the other'* [HDA FGD, *Kebele* B Amhara]

For peer review only

Discussion

It is recognized that comprehensive efforts at multiple levels are required to successfully increase facility delivery rates [36], this is what has occurred in the study sites. Previous interventions In Ethiopia, that have focused on access barriers at one level have not been successful [29]. We found that the drivers of behavior change in our study sites varied over time. One of the initial catalysts was the prohibition of TBAs. TBAs have been prohibited in several other African countries, but the policy has often encountered problems such as the ban being ineffectual due to enforcement issues, TBAs continuing their work underground, accessibility remaining a key barrier, and poor quality facilities limiting the effects [37-43]. Our data suggest that, in our study sites, the ban has been effective because it was coupled with increasing the awareness of alternative options, the HDA model allowing pregnant women to be identified and followed, the ambulance service improving accessibility, and facilities being perceived as beneficial in terms of clinical and social outcomes.

Prohibiting home births is controversial, and it has been argued that it infringes on personal choice and autonomy [39 40]. This is exemplified in a ruling in the European Court of Human Rights that regulations which make home births difficult to obtain violate the right to a private life [44 45]. On the other hand it has been argued that the restrictions implemented in several African countries are made in the interest of public health and are thus justified. Whatever view is taken, an important consideration is whether such bans and sanctions result in families hiding home deliveries, and subsequently reducing their care seeking. For example, in Burkina Faso sanctions and verbal abuse for those who did not attend services resulted in these families being too fearful to access services when they needed them [46].

In our study as families began to experience facility deliveries, the driver changed from push factors to the pull of a desire for facility deliveries. Positive experiences at the facility changed perceptions, and neighbors shared their experiences providing social proof. If families continue to have and share positive experiences the increase is likely to be sustained.

The importance of achieving saturation of messages is often overlooked within public health behavior change. Achieving high exposure has received less attention than the development high quality messages, yet high exposure appears to be more important for success [47 48]. The Ethiopian model allows high exposure to be achieved, as pregnant women can be effectively identified and followed by the HDA, and our data suggest this saturation was instrumental in increasing awareness and uptake of facility deliveries. Previous qualitative work in Ethiopia identified multiple barriers to facility delivery, including the normative nature of home deliveries, elders and neighbors pressing for a home delivery, misconceptions about facility deliveries, undesirable practices at facilities such as not allowing family in the delivery room, and poor treatment by health workers [13-20]. In our study none of these issues were reported suggesting that saturation of messages and positive experiences at the facility had resulted in perceptions being changed.

We found that elders had lost their decision making power, because their sons and daughters, due to information from the HEW, were now perceived as being more knowledgeable. There is often an assumption that elders are resistant to change, but we found, as others have [49], that their views can change rapidly given the right circumstances.

For peer review only - http://bmjopen.bmj.com/site/about/guidelines.xhtml

We found that different respondent groups highlighted different reasons for change. Efforts to make facilities friendly such as the provision of food and allowing family in the delivery room were particularly appreciated by grandmothers. Mothers reported more appreciation of medical interventions such as the use of injections. Husbands, who have been identified as making financial decisions regarding delivery location in other studies [50 51], were the only group to report on the impact of reduced costs and increased accessibility. Collecting data from multiple groups is important both for the design and evaluation of interventions.

We took several steps to maximize data quality, and took measures to improve the transferability of our findings including: using multiple study sites, purposive sampling to saturation, reflexivity, triangulation of methods and respondent groups and within and cross case analysis [52 53]. The study sites were all reasonably accessible and had reasonably functioning HEW systems. It is likely that distance and accessibility are the main factors influencing delivery location in less accessible areas, with our own respondents reporting that they knew of areas where women were unable to deliver in facilities. Studies in other settings in Ethiopia would further enhance transferability.

The main limitation of our data is the potential for social desirability bias given the pressure for women to deliver in a health facility – highlighted by the difficulty we had identifying women who delivered at home. The potential for social desirability bias has been identified as particularly high in Ethiopia given a political context that may limit how freely participants feel able to speak [35]. Although we used methods to help overcome such bias, respondents may still have been unwilling to say negative things about facility delivery, especially those identified for interview by the HEW/HDA. In addition, those respondents identified by the HEW/HDA may have been selected because of their positive attitudes. To try to reduce this we utilized snowball sampling to identify respondents where possible.

Our findings highlight the need to employ strategies that act at multiple levels, and that both push and pull families to health facilities. The unique administrative and political context of Ethiopia has facilitated behavior change, especially the ability to achieve saturation and penetration of messages and to identify and follow pregnant women. The increase is likely to be sustained if families' experiences of health facilities continue to be positive. Given the uniqueness context it is difficult to transfer findings to other countries where, for example, an HDA type network may not function as well. But, we feel that the key messages of focusing interventions at multiple levels, addressing pull and push factors and ensuring saturation of messages are useful for policy makers in other settings to consider.

For peer review only

For peer review only

References

1. Alkema L, Chou D, Hogan D, et al. Global, regional, and national levels and trends in maternal mortality between 1990 and 2015, with scenario-based projections to 2030: a systematic analysis by the UN Maternal Mortality Estimation Inter-Agency Group. Lancet (London, England) 2016;**387**(10017):462-74 doi: 10.1016/s0140-6736(15)00838-7[published Online First: Epub Date]|.

2. Filippi V, Ronsmans C, Campbell OM, et al. Maternal health in poor countries: the broader context and a call for action. Lancet (London, England) 2006;**368**(9546):1535-41 doi: 10.1016/s0140-6736(06)69384-7[published Online First: Epub Date]|.

3. Starrs AM. Safe motherhood initiative: 20 years and counting. Lancet (London, England) 2006;**368**(9542):1130-2 doi: 10.1016/s0140-6736(06)69385-9[published Online First: Epub Date]|.

4. World Health Organization. Skilled attendant at birth: 2006 updates. Geneva, 2006.

5. Demographic and Health Survey. STAT Compiler. Secondary STAT Compiler.

6. Federal Ministry of Health Planning and Programming Department. Health Sector Strategic Plan (HSDP-III), 2005/6-2009/10, 2005.

7. Teklehaimanot HD, Teklehaimanot A. Human resource development for a community-based health extension program: a case study from Ethiopia. Human resources for health 2013;**11**:39 doi: 10.1186/1478-4491-11-39[published Online First: Epub Date]|.

8. Banteyerga H. Ethiopia's health extension program: improving health through community involvement. MEDICC review 2011;**13**(3):46-9

9. Koblinsky M TF, Gaym A, Karim AM, Carnell M, Tesfaye S. Responding to the maternal health care challenge: The Ethiopian Health Extension Program. Ethiop J Health Dev 2010;**24**(Special Issue 1):105–09

10. National scale-up of integrated community case management in rural Ethiopia: implementation and early lessons learned. Ethiopian medical journal 2014;**52 Suppl 3**:15-26

11. Maes K, Closser S, Vorel E, et al. A Women’s Development Army: Narratives of Community Health Worker Investment and Empowerment in Rural Ethiopia. Studies in Comparative International Development 2015;**50**(4):455-78 doi: 10.1007/s12116-015-9197-z[published Online First: Epub Date]|.

12. Medhanyie A, Spigt M, Kifle Y, et al. The role of health extension workers in improving utilization of maternal health services in rural areas in Ethiopia: a cross sectional

For peer review only

study. BMC Health Services Research 2012;**12**(1):352 doi: 10.1186/1472-6963-12-352[published Online First: Epub Date]|.

13. Bedford J, Gandhi M, Admassu M, et al. 'A normal delivery takes place at home': a qualitative study of the location of childbirth in rural Ethiopia. Maternal and child health journal 2013;**17**(2):230-9 doi: 10.1007/s10995-012-0965-3[published Online First: Epub Date]|.

14. Gebrehiwot T, Goicolea I, Edin K, et al. Making pragmatic choices: women's experiences of delivery care in Northern Ethiopia. BMC pregnancy and childbirth 2012;**12**:113 doi: 10.1186/1471-2393-12-113[published Online First: Epub Date]|.

15. Gebrehiwot T, San Sebastian M, Edin K, et al. Health workers' perceptions of facilitators of and barriers to institutional delivery in Tigray, Northern Ethiopia. BMC pregnancy and childbirth 2014;**14**:137 doi: 10.1186/1471-2393-14-137[published Online First: Epub Date]|.

16. Jackson R, Tesfay FH, Godefay H, et al. Health Extension Workers' and Mothers' Attitudes to Maternal Health Service Utilization and Acceptance in Adwa Woreda, Tigray Region, Ethiopia. PloS one 2016;**11**(3):e0150747 doi: 10.1371/journal.pone.0150747[published Online First: Epub Date]|.

17. Roro MA, Hassen EM, Lemma AM, et al. Why do women not deliver in health facilities: a qualitative study of the community perspectives in south central Ethiopia? BMC research notes 2014;**7**:556 doi: 10.1186/1756-0500-7-556[published Online First: Epub Date]|.

18. Shifraw T, Berhane Y, Gulema H, et al. A qualitative study on factors that influence women's choice of delivery in health facilities in Addis Ababa, Ethiopia. BMC pregnancy and childbirth 2016;**16**(1):307 doi: 10.1186/s12884-016-1105-7[published Online First: Epub Date]|.

19. Sipsma H, Thompson J, Maurer L, et al. Preferences for home delivery in Ethiopia: provider perspectives. Global public health 2013;**8**(9):1014-26 doi: 10.1080/17441692.2013.835434[published Online First: Epub Date]|.

20. Zerihun A, Admassu M, Tulloch O, et al. Context analysis: Close-to community providers in ethiopia. Ethiopia, 2014.

21. Central Statistical Agency (CSA) [Ethiopia] and ICF. Ethiopia Demographic and Health Survey 2016: Key Indicators Report. Addis Ababa, Ethiopia, and Rockville, Maryland, USA, 2016.

22. Federal Democratic Republic of Ethiopia Ministry of Health. Health sector development programme iv annual performance report, EFY 2007 (2014/15), 2015.

23. The Federal Democratic Republic of Ethiopia Ministry of Health. Health Sector Transformation Plan 2015/16 - 2019/20 (2008-2012 EFY), 2015.

For peer review only

24. Federal Democratic Republic of Ethiopia Ministry of Health. Health sector transformation plan i. Annual performance report, EFY 2008 (2015/16), 2016.

25. Kok MC, Kea AZ, Datiko DG, et al. A qualitative assessment of health extension workers' relationships with the community and health sector in Ethiopia: opportunities for enhancing maternal health performance. Human resources for health 2015;**13**(1):80 doi: 10.1186/s12960-015-0077-4[published Online First: Epub Date]|.

26. Wang H, Tesfaye R, Gandham R, et al. Ethiopia Health Extension Program: An Institutionalized Community Approach for Universal Health Coverage. Washington D.C, 2016.

27. Banteyerga H. Boosting Maternal Health Care Seeking Behavior in Rural Low Income Communities: A Case Study of West Gojam and South Wollo Zones in Amhara, Ethiopia. American Journal of Health Research 2014;**2**(6):378-86

28. Godefay H, Kinsman J, Admasu K, et al. Can innovative ambulance transport avert pregnancy-related deaths? One-year operational assessment in Ethiopia. Journal of global health 2016;**6**(1):010410 doi: 10.7189/jogh.06.010410[published Online First: Epub Date]|.

29. Atnafu H, Belete Z, Kinfu H, et al. Can a community-based maternal care package in rural Ethiopia increase the use of health facilities for childbirth and reduce the stillbirth rate? International journal of women's health 2016;**8**:421-8 doi: 10.2147/ijwh.s104160[published Online First: Epub Date]|.

30. The Last Ten Kilometers. Improving maternal and newborn health care practices. The role of community based data for decision making News from the last ten kilometers 2013;**1**(3)

31. Betemariam B KA, Getachew N. Evidence based scaling-up an innovative community-based maternal and newborn health strategy in Ethiopia. 141st American Public Health Assocition annual meeting. Boston, USA, 2013.

32. Darmstadt GL, Marchant T, Claeson M, et al. A strategy for reducing maternal and newborn deaths by 2015 and beyond. BMC pregnancy and childbirth 2013;**13**:216 doi: 10.1186/1471-2393-13-216[published Online First: Epub Date]|.

33. Karim A. Effectiveness evaluation of a large-scale community-based program: Lessons from Ethiopia. Annals of Global Health;**81**(1):192 doi: 10.1016/j.aogh.2015.02.940[published Online First: Epub Date]|.

34. Michie S, van Stralen MM, West R. The behaviour change wheel: a new method for characterising and designing behaviour change interventions. Implementation science : IS 2011;**6**:42 doi: 10.1186/1748-5908-6-42[published Online First: Epub Date]|.

For peer review only

35. Ostebo MT, Cogburn MD, Mandani AS. The silencing of political context in health research in Ethiopia: why it should be a concern. Health Policy Plan 2018;**33**(2):258-70

36. Bohren MA, Hunter EC, Munthe-Kaas HM, et al. Facilitators and barriers to facility-based delivery in low- and middle-income countries: a qualitative evidence synthesis. Reproductive health 2014;**11**(1):71 doi: 10.1186/1742-4755-11-71[published Online First: Epub Date]|.

37. Godlonton S, Okeke EN. Does a ban on informal health providers save lives? Evidence from Malawi. Journal of development economics 2016;**118**:112-32 doi: 10.1016/j.jdeveco.2015.09.001[published Online First: Epub Date]|.

38. Turinawe EB, Rwemisisi JT, Musinguzi LK, et al. Traditional birth attendants (TBAs) as potential agents in promoting male involvement in maternity preparedness: insights from a rural community in Uganda. Reproductive health 2016;**13**:24 doi: 10.1186/s12978-016-0147-7[published Online First: Epub Date]|.

39. Rudrum S. Traditional Birth Attendants in Rural Northern Uganda: Policy, Practice, and Ethics. Health care for women international 2016;**37**(2):250-69 doi: 10.1080/07399332.2015.1020539[published Online First: Epub Date]|.

40. Sarelin A. Modernisation of Maternity Care in Malawi. Nordic Journal of Human Rights 2014;**32**(4):331-51 doi: 10.1080/18918131.2014.957463[published Online First: Epub Date]|.

41. Banda E. Stakeholders' perceptions of the changing role of traditional birth attendants in the rural areas of central west zone, Malawi: A mixed methods study. University of the Witwatersrand, 2013.

42. Rishworth A, Dixon J, Luginaah I, et al. "I was on the way to the hospital but delivered in the bush": Maternal health in Ghana's Upper West Region in the context of a traditional birth attendants' ban. Social science & medicine (1982) 2016;**148**:8-17 doi: 10.1016/j.socscimed.2015.11.018[published Online First: Epub Date]|.

43. Cheelo C, Nzala S, Zulu JM. Banning traditional birth attendants from conducting deliveries: experiences and effects of the ban in a rural district of Kazungula in Zambia. BMC pregnancy and childbirth 2016;**16**(1):323 doi: 10.1186/s12884-016-1111-9[published Online First: Epub Date]|.

44. Eggermont M. The choice of child delivery is a European human right. European journal of health law 2012;**19**(3):257-69

45. Stromerova Z. Midwifery in East Europe: Challenges and solutions. Midwifery Matters 2012(133):4-6

For peer review only

46. Melberg A, Diallo AH, Ruano AL, et al. Reflections on the Unintended Consequences of the Promotion of Institutional Pregnancy and Birth Care in Burkina Faso. PloS one 2016;**11**(6)

47. Hornik RC. Exposure: Theory and Evidence about All the Ways it Matters. Social Marketing Quarterly 2002;**8**(3):31-37 doi: 10.1080/15245000214135[published Online First: Epub Date]|.

48. Murray J, Remes P, Ilboudo R, et al. The Saturation+ Approach to Behavior Change: Case Study of a Child Survival Radio Campaign in Burkina Faso. Global health, science and practice 2015;**3**(4):544-56 doi: 10.9745/ghsp-d-15-00049[published Online First: Epub Date]|.

49. Aubel J, Toure I, Diagne M. Senegalese grandmothers promote improved maternal and child nutrition practices: the guardians of tradition are not averse to change. Social science & medicine (1982) 2004;**59**(5):945-59 doi: 10.1016/j.socscimed.2003.11.044[published Online First: Epub Date]|.

50. Dumbaugh M, Tawiah-Agyemang C, Manu A, et al. Perceptions of, attitudes towards and barriers to male involvement in newborn care in rural Ghana, West Africa: a qualitative analysis. BMC pregnancy and childbirth 2014;**14**:269 doi: 10.1186/1471-2393-14-269[published Online First: Epub Date]|.

51. Iganus R, Hill Z, Manzi F, et al. Roles and responsibilities in newborn care in four African sites. Tropical medicine & international health : TM & IH 2015;**20**(10):1258-64 doi: 10.1111/tmi.12550[published Online First: Epub Date]|.

52. Polit DF, Beck CT. Generalization in quantitative and qualitative research: myths and strategies. International journal of nursing studies 2010;**47**(11):1451-8 doi: 10.1016/j.ijnurstu.2010.06.004[published Online First: Epub Date]|.

53. Finfgeld-Connett D. Generalizability and transferability of meta-synthesis research findings. Journal of advanced nursing 2010;**66**(2):246-54 doi: 10.1111/j.1365-2648.2009.05250.x[published Online First: Epub Date]|.

For peer review only

Author contributions: ZH and JS conceived the study; ZH, YA and PA designed the study and the data collection tools; YA, PA and ZH were responsible for the acquisition of data. ZH, PS and YA were responsible for analysing and interpreting the data. ZH prepared the first draft of the paper and all authors commented on the paper and approved the final version.

For peer review only

For peer review only - http://bmjopen.bmj.com/site/about/guidelines.xhtml

Reporting checklist for qualitative study.

Based on the SRQR guidelines.

Instructions to authors

Complete this checklist by entering the page numbers from your manuscript where readers will find each of the items listed below.

Your article may not currently address all the items on the checklist. Please modify your text to include the missing information. If you are certain that an item does not apply, please write "n/a" and provide a short explanation.

Upload your completed checklist as an extra file when you submit to a journal.

In your methods section, say that you used the SRQR reporting guidelines, and cite them as:

O'Brien BC, Harris IB, Beckman TJ, Reed DA, Cook DA. Standards for reporting qualitative research: a synthesis of recommendations. Acad Med. 2014;89(9):1245-1251.

		Reporting Item	Page Number
	#1	Concise description of the nature and topic of the study identifying the study as qualitative or indicating the approach (e.g. ethnography, grounded theory) or data collection methods (e.g. interview, focus group) is recommended	4
	#2	Summary of the key elements of the study using the abstract format of the intended publication; typically includes background, purpose, methods, results and conclusions	2
Problem formulation	#3	Description and signifcance of the problem / phenomenon studied: review of relevant theory and empirical work; problem statement	3
Purpose or research question	#4	Purpose of the study and specific objectives or questions	3
Qualitative approach and research paradigm	#5	Qualitative approach (e.g. ethnography, grounded theory, case study, phenomenolgy, narrative research) and	3

For peer review only

		guiding theory if appropriate; identifying the research paradigm (e.g. postpositivist, constructivist / interpretivist) is also recommended; rationale. The rationale should briefly discuss the justification for choosing that theory, approach, method or technique rather than other options available; the assumptions and limitations implicit in those choices and how those choices influence study conclusions and transferability. As appropriate the rationale for several items might be discussed together.	
Researcher characteristics and reflexivity	#6	Researchers' characteristics that may influence the research, including personal attributes, qualifications / experience, relationship with participants, assumptions and / or presuppositions; potential or actual interaction between researchers' characteristics and the research questions, approach, methods, results and / or transferability	6
Context	#7	Setting / site and salient contextual factors; rationale	4
Sampling strategy	#8	How and why research participants, documents, or events were selected; criteria for deciding when no further sampling was necessary (e.g. sampling saturation); rationale	4
Ethical issues pertaining to human subjects	#9	Documentation of approval by an appropriate ethics review board and participant consent, or explanation for lack thereof; other confidentiality and data security issues	6
Data collection methods	#10	Types of data collected; details of data collection procedures including (as appropriate) start and stop dates of data collection and analysis, iterative process, triangulation of sources / methods, and modification of procedures in response to evolving study findings; rationale	4-6
Data collection instruments and technologies	#11	Description of instruments (e.g. interview guides, questionnaires) and devices (e.g. audio recorders) used for data collection; if / how the instruments(s) changed over the course of the study	5
Units of study	#12	Number and relevant characteristics of participants, documents, or events included in the study; level of	5

For peer review only

		participation (could be reported in results)	
Data processing	#13	Methods for processing data prior to and during analysis, including transcription, data entry, data management and security, verification of data integrity, data coding, and anonymisation / deidentification of excerpts	6
Data analysis	#14	Process by which inferences, themes, etc. were identified and developed, including the researchers involved in data analysis; usually references a specific paradigm or approach; rationale	6
Techniques to enhance trustworthiness	#15	Techniques to enhance trustworthiness and credibility of data analysis (e.g. member checking, audit trail, triangulation); rationale	6
Syntheses and interpretation	#16	Main findings (e.g. interpretations, inferences, and themes); might include development of a theory or model, or integration with prior research or theory	7-10
Links to empirical data	#17	Evidence (e.g. quotes, field notes, text excerpts, photographs) to substantiate analytic findings	7-10
Intergration with prior work, implications, transferability and contribution(s) to the field	#18	Short summary of main findings; explanation of how findings and conclusions connect to, support, elaborate on, or challenge conclusions of earlier scholarship; discussion of scope of application / generalizability; identification of unique contributions(s) to scholarship in a discipline or field	11-12
Limitations	#19	Trustworthiness and limitations of findings	12
Conflicts of interest	#20	Potential sources of influence of perceived influence on study conduct and conclusions; how these were managed	12
Funding	#21	Sources of funding and other support; role of funders in data collection, interpretation and reporting	1

The SRQR checklist is distributed with permission of Wolters Kluwer © 2014 by the Association of American Medical Colleges. This checklist can be completed online using https://www.goodreports.org/, a tool made by the EQUATOR Network in collaboration with Penelope.ai

For peer review only

BMJ Open

"People have started to deliver in the facility these days": A qualitative exploration of factors affecting facility delivery in Ethiopia

Journal:	*BMJ Open*
Manuscript ID	bmjopen-2018-025516.R1
Article Type:	Research
Date Submitted by the Author:	04-Feb-2019
Complete List of Authors:	Hill, Z; University College London, Institute for Global Health<br>Amare, Yared; Consultancy for Social Development<br>Scheelbeek, Pauline; University College London, Institute For Global Health<br>Schellenberg, Joanna; London School of Hygiene & Tropical Medicine, Dept of Infectious Disease Epidemiology
**Primary Subject Heading**:	Public health
Secondary Subject Heading:	Global health, Health services research, Qualitative research
Keywords:	facility delivery, qualitative, ethiopia, africa, community health workers

SCHOLARONE™
Manuscripts

"*People have started to deliver in the facility these days*": A qualitative exploration of factors affecting facility delivery in Ethiopia

Dr Zelee Hill (corresponding author), University College London, 30 Guilford Street, London WC1N 1EH, UK. E mail: z.hill@ucl.ac.uk

Dr Yared Amare, Consultancy for Social Development, PO Box 70196, Addis Ababa, Ethiopia. yaredamare@yahoo.com

Dr Pauline Scheelbeek, University College London, 30 Guilford Street, London WC1N 1EH, UK. pauline.scheelbeek@gmail.com

Professor Joanna Schellenberg, London School of Hygiene and Tropical Medicine, Keppel Street, London, WC1E 7HT, UK. joanna.schellenberg@lshtm.ac.uk

For peer review only

Abstract

Objectives: To understand the recent rise in facility deliveries in Ethiopia
Design: A qualitative study
Setting: Four rural communities in two regions of Ethiopia
Participants: 12 narrative, 12 in-depth interviews and four focus group discussions with recently delivered women; and four focus group discussions with each of grandmothers, fathers and community health workers.
Results: We found that several interwoven factors led to the increase in facility deliveries, and that respondents reported that the importance of these factors varied over time. The initial catalysts were a saturation of messages around facility delivery, improved accessibility of facilities, the prohibition of TBAs, and elders having less influence on deciding the place of delivery. Once women started to deliver in facilities, the drivers of the behavior changed as women had positive experiences. As more women began delivering in facilities, families shared positive experiences of the facilities, leading to others deciding to deliver in a facility.
Conclusion: Our findings highlight the need to employ strategies that act at multiple levels, and that both push and pull families to health facilities.

Key words: Facility delivery; Ethiopia, Qualitative

Word count: 4355

Article Summary: Strengths and limitations:

- The use of qualitative methods to understand a complex issue
- Use of multiple study sites, purposive sampling to saturation, reflexivity, triangulation of methods and respondent groups, and within and cross case analysis
- The study sites were all reasonably accessible, within half an hour walk from a motorable road, and had reasonably functioning Health Extension Worker systems. The study may thus underestimate accessibility issues, and the mechanism related to message saturation is unlikely to be triggered where Health Extension Workers function sub-optimally.
- There is the potential for social desirability bias given the pressure for women to deliver in a health facility, and because study mothers were mainly identified by Health Extension Workers or members of the Health Development Army.

Funding statement: This work was supported by the Bill and Melinda Gates Foundation as part of the IDEAS study

Competing interests: The authors have no competing interests

Data availability statement: Unpublished data from this study are not available. Data anonymity is not obtainable given the depth of the narrative qualitative transcripts, even when these have been anonymised.

Author contributions: Zelee Hill (ZH) and Joanna Schellenberg (JS) conceived the study. ZH, Yared Amare (YA) and Pauline Scheelbeek (PS) designed the data collection tools and strategy, YA and PS oversaw data collection, ZH, AY and PS conducted the analysis. ZH wrote the first draft of the manuscript and all authors contributed to the re-drafting and revision of the manuscript.

For peer review only

Introduction

Sub-Saharan Africa accounts for an estimated 66% of all maternal deaths.[1] Despite recent declines, mortality rates are still unacceptably high. Most deaths are preventable and occur around the time of delivery. Although it is essential to address the broad determinants of maternal mortality, such as female education and social status,[2] having a skilled attendant at delivery is nevertheless considered to be the most critical intervention.[3 4]

Ethiopia had one of the lowest levels of facility delivery coverage in the world, with Demographic and Health Survey (DHS) data showing coverage of 5% in 2000, 6% in 2005, and 11% in 2011 (three year recall).[5] This low coverage persisted despite government efforts to increase the accessibility and availability of services.[6 7] Efforts included the introduction of the Health Extension Program in 2003, where two female Health Extension Workers (HEW), stationed at newly constructed health posts, and paid around $100 a month, were trained to serve around 5,000 people. They provide health promotion, disease prevention and treatment, and work both at the health post and in the community.[8-11] The program had little impact on the coverage of skilled delivery, even when Health Extension Workers were trained to conduct deliveries in the health post,[7 12] and studies have found persistent and multiple barriers to change.[13-20]

By 2016 the DHS, using the same methodology to measure coverage as in previous surveys, found that coverage increased to 33% (3 year recall, with a regional range of 18-97%), a 200% increase from 2011.[21] The Ethiopian government attributes the increase largely to the work of the Health Development Army (HDA).[22-24] The HDA, created in 2012, is a network of all women in rural areas, organized into development groups of 30 women (1-30 networks), who are further clustered into groups of 6 (1-5 networks).[20 25-27] Groups select a leader who is then trained and supervised by the HEW. The HDA leaders help members adopt practices promoted by the HEW, hold participatory learning and action meetings, link pregnant women with care providers, hold monthly meetings for pregnant women, mobilize communities to contribute resources to make facilities mother friendly, and facilitate the use of either traditional or modern ambulances.[11 20 22 26]

Around the same time as the creation of the HDA, there were other policy changes that could have influenced facility delivery rates. These include the prohibition of the use of Traditional Birth Attendants (TBAs) for delivery,[20 25] and the provision of a four-wheel drive ambulance to transport women to facilities for delivery to every rural district.[28] In addition the number of health centers, staffed with two midwives, increased and the road infrastructure was also improved.[25 29] In this paper we use qualitative data to explore the reasons for the increase in facility deliveries in four study sites, we used a phenomenological approach as we were interested in understanding lived experiences.

For peer review only - http://bmjopen.bmj.com/site/about/guidelines.xhtml

Methods:

Study setting selection and characteristics: Data were collected between March and May 2015, from two wards (*Kebeles),* the smallest unit of local government, in the Southern Nations, Nationalities and Peoples region (SNNP) and two in Amhara region. Amhara has shown an increase in facility deliveries from 11% (2011) to 35% (2016), and SNNP region from 7% to 33%.[5]

Data were collected from areas where 'The Last Ten Kilometers' (L10K) programme was active in supporting the Health Extension Program. *Kebeles* were selected from a list, provided by L10K project staff, of *kebeles* considered to have a reasonably functioning HEW system, that is that they had HEWs in place that were considered to be active and working well. Other selection criteria were that the *kebeles* were seen as typical of the district (*Woreda*) with no unusual characteristics such as having a large hospital or a large industry close by, and were less than half an hours walk from a motorable road so that the study team could feasibly access them. We have labeled these *kebele* "A-D" to maintain anonymity. Table 1 shows the characteristics of the selected *kebele*s, all of which had a subsistence farming based economy. Although the study sites were all a short walk from a motorable road, access to public transport was very limited.

Table 1. Characteristics of Study *kebele*

Region	*kebele*	Ethnicity	Predominant religion	Access to health centers	Terrain
Amhara	*kebele* A	Amhara	Orthodox Christian	Moderate	Hilly
	kebele B	Amhara	Orthodox Christian	High	Hilly
SNNPR	*kebele* C	Gamo/ Wolaita	Protestant/ Orthodox Christian	High	Predominantly flat with some hilly parts
	kebele D	Silte	Muslim	Moderate	Flat

Data collection: Data were collected as part of a study to understand how HEWs influence maternal and newborn care behaviors, of which facility delivery was one. Four trained interviewers collected data in the local language using pre-tested semi structured guides developed by the authors. When needed translators were used. The content of the guides was informed by a theoretical framework, which identified pathways through which HEWs could influence behaviors by modifying families capabilities, opportunities and motivation.[30] Data were collected from mothers, grandmothers, fathers, HEWs and HDA using narrative interviews, in-depth interviews, and focus group discussions. All community respondents had children or grandchildren under 12 months of age, with narrative mothers having children less than three months of age to facilitate recall. Using a range of both methods and respondents allowed for data triangulation and ensured we captured a range of viewpoints. Narrative interviews with mothers were used to capture personal experiences, in-depth interviews to capture perceptions of what was commonly done in the community, and focus group discussions to collect data that we felt would benefit from being discussed in a group interaction.

For peer review only - http://bmjopen.bmj.com/site/about/guidelines.xhtml

Data were collected until saturation was reached, i.e. until additional interviews provided similar information to that already obtained. Saturation was determined by frequent transcript reviews. The sample size, respondent groups, and the interview content related to facility delivery are shown in Table 2. In the FGD we employed several activity oriented exercises such as sorting and ranking and root cause analysis to encourage group interaction and participation and reduce social desirability bias, which can be a particular issue in Ethiopia.[31]

For peer review only

Table 2: Data collection method, sample size and content related to facility delivery

Method	Sample	Interview content related to facility delivery
Narrative interviews with recent mothers	12	• Labor and delivery story<br>• Perceived knowledge and skills related to pregnancy and newborn care<br>• Description of contacts during pregnancy and delivery with health workers, HDAs and HEWs<br>• Information received on where to deliver, source of the information, reaction to the information, and the impact of the information on decision making
In-depth interviews with recent mothers	13	• Perceptions of where most people deliver, and community views of those who deliver at home and those who deliver in a facility<br>• Views on HEW/HDA work and their suitability<br>• Description of contacts during pregnancy and delivery with health workers, HDAs and HEWs<br>• Information received on where to deliver, source of the information and reaction to the information<br>• Most significant maternal and newborn health changes in the community in the last 2 years, and why things changed
FGD with recent mothers	4	• Pile sort of behaviors practiced/not practiced, important/not important, and that are promoted/not promoted by HEWs/HDAs<br>• Community views of those who deliver at home and those who deliver in a facility<br>• Most significant maternal and newborn health changes in the last 2 years, and why things changed<br>• Reaction to statements that HEWs/HDAs work does not bring change, and that people dislike HEWs telling them where to deliver
FGD with grand-mothers	4	• Reaction to a picture of a facility delivery<br>• Most significant maternal and newborn health changes in the last 2 years and why things changed<br>• Reaction to statements about grandmothers supporting traditional practices, and that mothers do not listen to grandmother advice
FGDs with fathers	4	• Reaction to a picture of a facility delivery<br>• Fathers role in deciding place of delivery<br>• Response to a scenario where a family does not follow HEW advice<br>• Views on HEW/HDA work and their suitability<br>• Most significant maternal and newborn health change in the last 2 years and why things changed<br>• Reaction to statements that mothers/fathers make decisions about delivery, and that people dislike HEWs telling them where to deliver
FGD with HEW and HDA	4	• Pile sort of behaviors practiced/not practiced, important/not important, and that are promoted/not promoted by HEWS/HDAS<br>• Most significant changes in the community, and in their work, related to maternal and newborn health in the last 2 years and why things changed<br>• Successes and challenges they faced in encouraging behavior

For peer review only

		change<br>• Reaction to statements that mothers get punished if they do not follow advice, and that mothers prefer advice of family members

Mothers, grandmothers and fathers, from different households, were identified by the HEW/HDA, or through snowball sampling from the community respondents – with the first method providing the majority of respondents. Eligibility criteria were that the family had received at least one visit by an HEW or HDA. Mothers were selected to ensure diversity in age, educational level, parity, sex of newborn and socio-economic status. Grandmothers could be paternal or maternal – as long as they had been dependent on which was closest to the family. We also aimed to get diversity in place of delivery, but located few women who admitted they delivered at home. All of the HDAs in the study *kebeles* were invited for the HDA FGDs. As there were only 2 HEWs per *kebele* HEW FGDs included HEWs from neighboring *kebeles.* Interviewers approached potential respondents in their home, or at the health post. Three respondents refused, as they were too busy. Interviews lasted from 1 to 2 hours and took place in respondents' houses, or the health post for the HEW. FGDs were conducted with 3-7 *respondents* in neutral locations and lasted from 1.5 to 2.5 hours. HEWs and HDAs were not present during any of the interviews or FGDs with community members.

Interviews and FGDs were audio-recorded and fully transcribed by the data collectors in English as soon as possible. Data collectors met regularly during fieldwork to discuss emerging themes and to receive feedback from the senior researchers. Ethical approval was granted by the Ministry of Science and Technology in Ethiopia and the London School of Hygiene and Tropical Medicine in the UK. Written informed consent was obtained from all respondents. On entering a household the interviewer introduced themselves and the project to key people, and gave the head of household a project leaflet. They explained who they wanted to interview read aloud a study information sheet to them in a quiet place. For FGDs the information was read aloud to all FGD respondents. The interviewers checked respondents' comprehension, rephrased if necessary and gave the respondents an opportunity to ask questions. If the respondent agreed to be interviewed the interviewer read the consent form out loud and asked the respondent to sign to show that they were willing to be interviewed, understood the study, were happy for their words to be written down and recorded, were happy for their quotes to be used, and for the information collected to be transferred to London. The interviewers also signed each form.

Respondent and public involvement: Respondents were not directly involved in the design of the study, however the interview guides were iterative and were modified as the research progressed based on reported experiences and perceptions. Some respondents were recruited through snowball sampling, i.e. where respondents suggested others they knew who were eligible for interview.

Data analysis: Analysis began during data collection through regular team meetings and reflection. A formal analysis session was held with the data collectors in the middle and at the end of data collection, this included discussion of how our characteristics could have influenced how data were collected and interpreted. Once data were collected all transcripts were read several times to ensure familiarity with the data, to begin to identify notable constructs and to see the data as a whole. A deductive coding template was developed in Nvivo based on the theoretical framework that guided the interview content. Interviews and focus groups were then coded inductively within these broad themes. Coding was done by identifying the underlying meaning of each section of text and how it was different or similar

For peer review only

to others section. Codes that contained similar concepts were then put into larger themes. Themes and codes were modified by looking for patterns, links and contradictions within themes. Data credibility was checked by triangulating data between respondent groups and between data collection methods. Data analysis was done by three of the senior researchers, who discussed their coding regularly to enhance conceptual thinking and to increase coding rigor. Reflective notes were kept throughout the process.

For peer review only

For peer review only - http://bmjopen.bmj.com/site/about/guidelines.xhtml

Results

Table 3 shows the characteristics of the narrative and IDI respondents. Respondents had a range of ages, education levels, parities, and religion. We did not achieve the planned diversity in place of delivery, as 19/25 of the narrative/IDI women had delivered in a facility. This is possibly because families were reluctant to admit to home deliveries, and because facility delivery rates may have been high in the study area because the sites were relatively accessible, within walking distance of a motorable road, and had functioning HEW systems. In addition HEW/HDAs assisted in identifying respondents, and may have favored those who delivered in a facility.

The FGD participant mothers were varied in age (range 19-35 years of age), parity (range 1-7 children), education (none-secondary level), and ethnicity. The FGD participant fathers were older (range 28-45 years of age) and the FGD participant grandmothers were less educated with almost all being uneducated. Grandmothers were predominantly, but not exclusively, paternal.

Table 3: Sample characteristics (narrative and mother IDIs)

Characteristic	Frequency (n=25)
**Age**	
≤ 24	10
25-34	10
≥35	5
**Education**	
None	10
Primary	12
Secondary and above	3
**Religion**	
Islamic	8
Christian	17
**Parity**	
1	7
2-3	7
≥4	11
**Place of last delivery**	
Home	6
Facility	19

All respondent groups reported that the increase in facility delivery was recent, and that previous attempts to encourage facility delivery had limited success: '*People have started to deliver in the facility these days……. They* [families] *used to give us lots of excuses like, let the cattle return back home, let the sun start setting, and let's wait for this and that; believing that the mother would deliver in the meantime….so that used to be very problematic'* [HEW FGD, *Kebele* A Amhara]. At the time of data collection, delivering in a health center was reported as the usual practice in all study communities, and respondents reported that: *'everyone knows what to do'… 'no one delivers at home'*.

For peer review only - http://bmjopen.bmj.com/site/about/guidelines.xhtml

Respondents reported that the factors that influenced facility delivery changed over time and consisted of push and pull factors. We identified four themes around the initial uptake of facility delivery: saturation of messages around facility delivery, improved accessibility of facilities, the prohibition of TBAs, and elders having less influence on deciding the place of delivery. Themes around the drivers of facility delivery once uptake had begun were around families having positive experiences of facilities, seeing the worth of a facility delivery, and sharing their positive experiences with others.

Saturation of messages and contacts: All mothers interviewed reported receiving information on the importance of facility delivery, and all respondent groups knew that it was being strongly promoted at: *'every opportunity'.* In the majority of cases mothers had received information from at least two sources, and at several time points: *'*[HDA] *tells me about it repeatedly and forcefully'* [Mother IDI, *Kebele* D SNNPR]. Information was mainly given at the health post, at home, during ANC, and at community meetings. The main sources of information were the HEWs and HDAs. The resultant high awareness levels was reported as a reason for the increase in facility delivery rates:

> *Interviewer: Why didn't you go* [to the facility] *at that time* [for previous deliveries]*?*
> *Respondent: Because there was no one who educates you like this at that time. Nobody advised us to deliver in the health center…… My knowledge was not as strong at that time* [Mother IDI, *Kebele* D SNNPR]

A theme that emerged from all respondent groups, related to how the information on facility delivery was received, was around community trust in health workers and HEWs. Trust in HEWs arose from a view that HEWs were knowledgeable because of their training and were higher status than community members: *'They* [HEW] *are better than us; they teach us what they have learned. She* [HEW] *went there* [training] *so that she could bring us some good education, we don't believe she teaches us harmful advice'* [Mother FGD, *Kebele* D SNNPR]. The theme around trust in HEWs was contrasted by views of the had leaders who were less trusted as they were viewed as people who transferred messages rather than being knowledgeable in their own right. But, HDA leaders played a key role in ensuring the penetration of messages, and by informing HEWs about pregnant women: *'The leaders of this group* [1-5 HDA group] *follow how many of them are pregnant …. The leaders know everything about their group…… And when labor starts, the leader will inform the HEWs'* [Mother IDI, *Kebele* B Amhara].

Improved accessibility: Knowledge of the ambulance service was universal across respondent groups. In some sites families were given the ambulance number during pregnancy. In other sites families called the HEW at the start of labor, and the HEWs then called the ambulance. The presence of the ambulance was reported as facilitating facility delivery by all groups:

> *'If ambulance service had not started functioning in the kebele, the mother surely gives birth at home'* [Mother FGD, *Kebele* A Amhara]
>
> *'This time there is no one who delivered at home, it was in our fathers' time, now there is ambulance which take the mother to the health center, so all women deliver there'* [Mother FGD, *Kebele* A Amhara]

A theme among fathers was the role that the increase in the number of health centers, free delivery care and the construction of roads had impacted on facility delivery uptake: *'Formerly people think there is a payment for delivery, like they pay for treatment but there*

For peer review only - http://bmjopen.bmj.com/site/about/guidelines.xhtml

is no such things … there were a lot of people who deliver at home thinking it [facility delivery] *needs money… even if she* [mother] *asked to go the husband didn't want, thinking he will be asked for money'* [Father FGD, *Kebele* D SNNPR].

Despite the reported importance of ambulances, several families interviewed described a problem accessing the service. This was most frequently because the ambulance was busy, could not come because of heavy rain, had no fuel, or took too long to come: *'I asked* [HEW] *to call an ambulance, but there was no ambulance so we were told to use public transport'* [Grandmother FGD, *Kebele* B Amhara]. In the narrative interviews over half of the women (6/9) who called an ambulance had a problem accessing the service, of these 3 delivered at home or with the HEW, 1 delivered in the health center but waited a long time for the ambulance and the other 2 took public transport.

The four study sites were reasonably accessible and respondents talked of villages where health facility delivery was still very difficult because of accessibility issues: '*Not all villages are accessible, those who live in X village, they are not able to deliver in health facility. But those who live in nearer villages….. It is a must to deliver in health facility since they are close'* [Mother IDI, *Kebele* A Amhara].

Prohibition of TBAs: In all sites the use of traditional birth attendants was reported as forbidden, with a threat of sanctions for those who conducted or had a home delivery:

> *During pregnancy, they* [HEW] *told me not to deliver at home. They said "if you deliver at home, you will be punished"….. 500 birr* [$22] *if I deliver at home* [Mother IDI, *Kebele* C SNNPR]
>
> *….birth attendants are not willing to assist due to fear of punishment….. the women herself will pay 2000 birr* [$88] *and the birth attendant will pay 1000 birr* [$44] [Mother IDI, *Kebele* B Amhara]

Decisions about sanctions were made at community level through the 1-5 or 1-30 HDA networks: *'The community decided a 'Sera'* [customary law] *that if a mother delivers at home she will be fined 500 birr* [$22]*'* [Narrative interview, *Kebele* B Amhara]. The reported sanctions were varied, with the fines ranging from 200-2000 birr [$9-88].

The possibility of being sanctioned for delivering at home was a key theme relating to the initial uptake of messages around facility deliveries among all respondent groups:

> Interviewer: *What do you think brought this change*?
> Respondent:…. *the fear of the punishment, I don't think most of the community understood the benefit of delivering in the facility…. didn't give due attention to the lessons….. it is after we are told we will be punished* [Mother IDI, *Kebele* C SNNPR]
>
> *Respondent 2: There is 1 to 5* [HDA group]*, and one watch over the other, and there is also punishment; if the mother deliver at home she will be fined with 500 birr* [$22].
> *Respondent 3: There is a law they are fined …. after that people start saying hurry up please she is going to deliver* [laughing] [Father FGD, *Kebele* C SNNPR]

For peer review only - http://bmjopen.bmj.com/site/about/guidelines.xhtml

Respondents, including former TBAs, reported that the sanctions were justified and beneficial as times were changing. HEWs were aware of them, and at times encouraged their use:

> *Respondent: When a mother delivers at home and if the baby dies….the government will prosecute her for that….. We use such threats.*
> *Interviewer: Who tells them such threats?*
> *Respondent: We call the HDA leaders* [1 to 30] *and then tell them that such threats may work, and then they go and tell the mothers* [HEW FGD, *Kebele* C SNNPR]

In only one site were there reports that sanctions had been used in practice, and the respondents that we interviewed who had delivered at home reported that they had been excused the sanctions: '[HEW said] *If you were another person I will take you to jail but you face a lot of problem that's why I left you'* [IDI mother, *Kebele* D SNNPR]. Although the respondents who delivered at home reported that sanctions were not applied, they did report that the HEWs were angry with them and, in a few cases, denied them services: *'She* [HEW] *suspected that I hid and delivered at home….. and was very angry. Because she was angry then, she did not tell me things…..there was no mention of how I should be bathing the baby and the like'* [Narrative woman, *Kebele* B Amhara].

Power shift: Grandmothers had little influence on place of delivery, and respondents in all groups used words like: *'we are in a different time'* and *'time has changed'.* This lack of influence was attributed to mothers being modern because of the education given by the HEW, were thus more knowledgeable than their elders, and were consequently able to challenge their advice: *Today's mothers are young and modern. They easily accept new ideas……. they wouldn't like to do the traditional practice ….. since they have received the new education* [Narrative mother, *Kebele* C SNNPR]. Husbands were viewed as having the ultimate decision making power in the household and generally supported facility delivery. This support from husbands put mothers in a stronger position if they faced opposition from their elders. We found very few grandmothers who reported that they were resistant to the change in delivery location.

Positive experiences: The main theme around how the drivers of facility delivery changed over time was around the influence of families having and sharing positive experiences of facility delivery:

> *I felt very happy* [to deliver in the facility]…. *The doctors give morale, they said take it easy, be strong and the like'* [Mother IDI, *Kebele* D SNNPR].

> *But now, it is not that they* [families] *are afraid of the punishment, they have started saying that they are going because they want to get care from the health professionals…. They have started saying that the physicians do all they can and help them deliver* [HDA FGD, *Kebele* D SNNPR]

> *Formerly they feared the penalty, but now those who delivered there* [at health center] *talk about good thing of delivering there* [Mother IDI, *Kebele* C SNNPR]

All respondents in all groups reported that facility delivery was safer and reduced deaths. The provision of an injection to stop bleeding was the most frequently mentioned benefit along with getting a vaccination for the baby, removal of dirt from the abdomen/stomach, the baby being cared for and not left alone, the facility being hygienic, delivery being less

For peer review only - http://bmjopen.bmj.com/site/about/guidelines.xhtml

painful and more predictable, and that the facility could deal with problems such as the baby being in the wrong position, the placenta getting stuck, or the baby being born weak:

> *I know that I will not be hurt if I deliver in the health center….. they will inject me something which stopped the excessive bleeding; I know that they will assist me if I will have any complications* [Mother IDI, *Kebele* C SNNPR]

> *Here* [home] *there is only suffering until the delivery nothing else, and we are delivering in the facility in a very relaxed way…… they* [health workers] *measure and tell us how much time is left……but here we don't know anything, we are just laboring and waiting until we deliver or die* [Mother IDI, *Kebele* D SNNPR]

All respondent groups reported that at the facility mothers received food, drink and sometimes a cloth for the baby: *'Everything is perfect, even porridge and gruel is prepared and served to mothers in the health center. It is really good…….. Even the person who accompanied them is invited'* [Grandmother FGD, *Kebele* B Amhara]. A theme related to grandmothers was that becasue they were allowed in the delivery room they had less fears about what occurred during a facility delivery: *'We* [health workers] *never used to allow anybody inside the delivery … as they* [family members] *start to see; they started saying 'we were afraid that you would insert materials inside her' …. they see that things are good'* [HEW FGD, *Kebele* A Amhara].

Not only did respondents report benefits and good experiences of delivering at the facility, they often shared their experience with their friends and neighbors, and several mothers reported that their friends and neighbors had influenced their delivery location:

> *I heard from other people, I heard that it is good to deliver in the health center, so I was planning to deliver there…. one of my neighbors delivered in the health facility, and she told me it is good…. She told me the mother will be very clean, she will not have bleeding….. health professionals would help the mother and save her life*. [Mother narrative, *Kebele* C SNNPR]

> *Respondent 1: Now, the unwilling ones also went there because she saw when others do.*
> *Respondent 2: Yes, formerly, they did not want to be exposed. They said that, Saint Mary will do what she wants. But now, they see the benefit. And learn one from the other'* [HDA FGD, *Kebele* B Amhara]

For peer review only - http://bmjopen.bmj.com/site/about/guidelines.xhtml

Discussion

A systematic review of qualitative studies exploring facility delivery classified the findings based on the quality and coherence of studies.[32] Barriers to facility delivery in which there was high confidence were: cultural barriers, such as perceptions of birth as a natural event; decision making barriers, including the role of elder women; proximity, access and cost barriers; a reliance on TBAs; and barriers related to perceived poor quality of care and mistreatment by health workers. High confidence facilitators were valuing facilities for complications and perceiving them as providing high quality of care. Previous birth experiences were both a barrier and a facilitator. Previous studies specific to Ethiopia identified similar barriers and.[13-20] In our study none of these barriers were reported, with the exception of accessibility issues for more remote villages. Our findings suggest that these barriers were overcome through a combination of saturation of messages around facility delivery from trusted sources, reduction in access issues, the prohibition of TBAs, power shifts away from grandmothers and positive experiences. The focus of this paper on what has driven the change process adds new insights to the literature, which to date has focused on barriers and facilitators to uptake rather than mechanisms of change. It is widely recognized that comprehensive efforts, at multiple levels, are required to successfully increase facility delivery rates,[32] this is what has occurred in the study sites. Previous interventions In Ethiopia, that have focused on access barriers at one level have not been successful.[29]

Respondents reported that the drivers of behavior change in our study sites varied over time. One of the initial catalysts was the prohibition of TBAs. TBAs have been prohibited in several other African countries, but the policy has often encountered problems such as the ban being ineffectual due to enforcement issues, TBAs continuing their work underground, accessibility remaining a key barrier, and poor quality facilities limiting the effects.[33-39] Our data suggest that, in our study sites, the ban has been effective. This could be because the ban was coupled with increased awareness of and access to alternative options, that were viewed positively, and because the HDA model allowing pregnant women to be identified and followed. We were unable to locate details of the TBA ban, but our data suggest that the specifics of the ban, in relation to whether and how it was implemented, were determined at local levels. There may be considerable variation in implementation and impact in other Ethiopian settings.

Prohibiting home births is controversial, and it has been argued that it infringes on personal choice and autonomy.[35 36] This is exemplified in a ruling in the European Court of Human Rights that regulations which make home births difficult to obtain violate the right to a private life.[40 41] On the other hand it has been argued that the restrictions implemented in several African countries are made in the interest of public health and are thus justified. Whatever view is taken, an important consideration is whether such bans and sanctions result in families hiding home deliveries, and subsequently reducing their care seeking. For example, in Burkina Faso sanctions and verbal abuse for those who did not attend services resulted in these families being too fearful to access services when they needed them.[42]

In our study as families began to experience facility deliveries, the driver changed from push factors to the pull of a desire for facility deliveries. Positive experiences at the facility changed perceptions, and neighbors shared their experiences. If families continue to have and share positive experiences the increase is likely to be sustained.

For peer review only - http://bmjopen.bmj.com/site/about/guidelines.xhtml

Saturation of messages was a key driver in increasing awareness and uptake of facility deliveries. The importance of achieving saturation of messages is often overlooked within public health behavior change, and achieving high exposure has received less attention than the development of high quality messages, yet high exposure appears to be equally important for success.[43 44] The HEW and HDA model, which was functional in our study sites, facilitated high exposure as pregnant women could be identified and followed.

We found that elders had lost their decision making power, because their sons and daughters, due to information from the HEW, were now perceived as being more knowledgeable. There is often an assumption that elders are resistant to change, but we found, as others have,[45] that their views can change rapidly in some circumstances.

We found that different respondent groups highlighted different reasons for change. Efforts to make facilities friendly such as allowing family in the delivery room were particularly appreciated by grandmothers while husbands, who have been identified as making financial decisions regarding delivery location in other studies,[46 47] were the only group to report on the impact of reduced costs, improved roads and a greater number of health centers. Collecting data from multiple groups is important both for the design and evaluation of interventions.

Data quality and study limitations: We took several steps to maximize data quality, and took measures to improve the transferability of our findings including: using multiple study sites, purposive sampling to saturation, reflexivity, triangulation of methods and respondent groups and within and cross case analysis.[48 49] Despite this the findings may not apply to other areas with significantly different contextual issues. For example the study sites were all reasonably accessible and had reasonably functioning HEW systems. It is likely that distance and accessibility are the main factors influencing delivery location in less accessible areas, with our respondents reporting that they knew of areas where women were unable to deliver in facilities because of distance. Studies in other settings in Ethiopia would further enhance transferability, however, the study findings suggest several issues that could be considered when exploring issues related to facility delivery coverage and the effectiveness of interventions to increase facility delivery rates in other settings.

The main limitation of our data is the potential for social desirability bias given the pressure for women to deliver in a health facility – highlighted by the difficulty we had identifying women who delivered at home. The potential for social desirability bias has been identified as particularly high in Ethiopia given a political context that may limit how freely respondents feel able to speak.[31] Although we used methods to help overcome such bias, respondents may still have been unwilling to say negative things about facility delivery, especially those identified for interview by the HEW/HDA. In addition, those respondents identified by the HEW/HDA may have been selected because of their positive attitudes and experiences. To try to reduce this we utilized snowball sampling to identify respondents, but the majority of the respondents were identified through the HEWs/HDAs. As a result, the study respondents may have had different attitudes and experiences to families that were less favored by the HEW/HDA.

Our findings highlight the need to employ strategies that act at multiple levels, and that both push and pull families to health facilities. The ability to achieve saturation and penetration of messages and to identify and follow pregnant women was a key factor in increasing facility deliveries, this is likely to have been influenced by the unique administrative and political context of Ethiopia. The increase is likely to be sustained if families' experiences of health

For peer review only - http://bmjopen.bmj.com/site/about/guidelines.xhtml

facilities continue to be positive and effort to improve the accessibility and quality of care continue; such as the provision and maintenance of ambulances, allowing family and cultural ceremonies into the delivery room, and the provision of food at the facility. Given the unique context it is difficult to transfer findings to other countries where, for example, an HDA type network may not function as well. But, we feel that the key messages of focusing interventions at multiple levels, addressing pull and push factors and ensuring saturation of messages are useful for policy makers in other settings to consider.

For peer review only

References

1. Alkema L, Chou D, Hogan D, et al. Global, regional, and national levels and trends in maternal mortality between 1990 and 2015, with scenario-based projections to 2030: a systematic analysis by the UN Maternal Mortality Estimation Inter-Agency Group. *Lancet* 2016;387(10017):462-74 doi: 10.1016/s0140-6736(15)00838-7.

2. Filippi V, Ronsmans C, Campbell OM, et al. Maternal health in poor countries: the broader context and a call for action. *Lancet* 2006;368(9546):1535-41 doi: 10.1016/s0140-6736(06)69384-7.

3. Starrs AM. Safe motherhood initiative: 20 years and counting. *Lancet* 2006;368(9542):1130-2 doi: 10.1016/s0140-6736(06)69385-9.

4. World Health Organization. Skilled attendant at birth: 2006 updates. Geneva:World Health Orghanization 2006.

5. ICF, 2012. The DHS Program STATcompiler. http://www.statcompiler.com. [Accessed December 2018].

6. Federal Ministry of Health Planning and Programming Department. Health sector strategic plan (HSDP-III), 2005/6-2009/10. Addis Ababa, Ethiopia: Federal Ministry of Health 2005.

7. Teklehaimanot HD, Teklehaimanot A. Human resource development for a community-based health extension program: a case study from Ethiopia. *Hum Resour Health* 2013;11:39 doi: 10.1186/1478-4491-11-39.

8. Banteyerga H. Ethiopia's health extension program: improving health through community involvement. *MEDICC Rev* 2011;13(3):46-9.

9. Koblinsky M, Tain F, Gaym A, et al. Responding to the maternal health care challenge: the Ethiopian health extension program. *Ethiop J Health Dev* 2010;24(Special Issue 1):105–09.

10. Legesse H, Degefie T, Hiluf M, et al. National scale-up of integrated community case management in rural Ethiopia: implementation and early lessons learned. *Ethiop Med J* 2014;52 Suppl 3:15-26

11. Maes K, Closser S, Vorel E, et al. A women's development army: narratives of community health worker investment and empowerment in rural Ethiopia. *Stud Comp Int Dev* 2015;50(4):455-78 doi: 10.1007/s12116-015-9197-z.

12. Medhanyie A, Spigt M, Kifle Y, et al. The role of health extension workers in improving utilization of maternal health services in rural areas in Ethiopia: a cross sectional study. *BMC Health Serv Res* 2012;12(1):352 doi: 10.1186/1472-6963-12-352.

For peer review only

13. Bedford J, Gandhi M, Admassu M, et al. 'A normal delivery takes place at home': a qualitative study of the location of childbirth in rural Ethiopia. *Matern Child Health J* 2013;17(2):230-9 doi: 10.1007/s10995-012-0965-3.

14. Gebrehiwot T, Goicolea I, Edin K, et al. Making pragmatic choices: women's experiences of delivery care in Northern Ethiopia. *BMC Pregnancy Childbirth* 2012;12:113 doi: 10.1186/1471-2393-12-113.

15. Gebrehiwot T, San Sebastian M, Edin K, et al. Health workers' perceptions of facilitators of and barriers to institutional delivery in Tigray, Northern Ethiopia. *BMC Pregnancy Childbirth* 2014;14:137 doi: 10.1186/1471-2393-14-137.

16. Jackson R, Tesfay FH, Godefay H, et al. Health extension workers' and mothers' attitudes to maternal health service utilization and acceptance in Adwa Woreda, Tigray Region, Ethiopia. *PLoS One* 2016;11(3):e0150747 doi: 10.1371/journal.pone.0150747.

17. Roro MA, Hassen EM, Lemma AM, et al. Why do women not deliver in health facilities: a qualitative study of the community perspectives in south central Ethiopia? *BMC Res Notes* 2014;7:556 doi: 10.1186/1756-0500-7-556.

18. Shifraw T, Berhane Y, Gulema H, et al. A qualitative study on factors that influence women's choice of delivery in health facilities in Addis Ababa, Ethiopia. *BMC Pregnancy Childbirth* 2016;16(1):307 doi: 10.1186/s12884-016-1105-7.

19. Sipsma H, Thompson J, Maurer L, et al. Preferences for home delivery in Ethiopia: provider perspectives. *Glob Public Health* 2013;8(9):1014-26 doi: 10.1080/17441692.2013.835434.

20. Zerihun A, Admassu M, Tulloch O, et al. Report on the context analysis: close-to community providers in Ethiopia, Hawassa, Ethiopia: Reach Out 2014.

21. Central Statistical Agency (CSA) [Ethiopia] and ICF. Ethiopia Demographic and Health Survey 2016: key indicators report. Addis Ababa, Ethiopia, and Rockville, Maryland, USA: CSA and ICF 2016.

22. Federal Democratic Republic of Ethiopia Ministry of Health. Health sector development programme IV annual performance report, EFY 2007 (2014/15). Addis Ababa, Ethiopia: Federal Ministry of Health 2015.

23. The Federal Democratic Republic of Ethiopia Ministry of Health. Health Sector Transformation Plan 2015/16 - 2019/20 (2008-2012 EFY). Addis Ababa, Ethiopia: Federal Ministry of Health 2015.

24. Federal Democratic Republic of Ethiopia Ministry of Health. Health sector transformation plan I. Annual performance report, EFY 2008 (2015/16). Addis Ababa, Ethiopia: Federal Ministry of Health 2016.

For peer review only

25. Kok MC, Kea AZ, Datiko DG, et al. A qualitative assessment of health extension workers' relationships with the community and health sector in Ethiopia: opportunities for enhancing maternal health performance. *Hum Resour Health* 2015;13(1):80 doi: 10.1186/s12960-015-0077-4.

26. Wang H, Tesfaye R, Ramana G, et al. Ethiopia health extension program: an institutionalized community approach for universal health coverage. Washington D.C, USA: World Bank 2016.

27. Banteyerga H. Boosting maternal health care seeking behavior in rural low income communities: a case study of West Gojam and South Wollo Zones in Amhara, Ethiopia. *American Journal of Health Research* 2014;2(6):378-86.

28. Godefay H, Kinsman J, Admasu K, et al. Can innovative ambulance transport avert pregnancy-related deaths? One-year operational assessment in Ethiopia. *J Glob Health* 2016;6(1):010410 doi: 10.7189/jogh.06.010410.

29. Atnafu H, Belete Z, Kinfu H, et al. Can a community-based maternal care package in rural Ethiopia increase the use of health facilities for childbirth and reduce the stillbirth rate? *Int J womens Health* 2016;8:421-8 doi: 10.2147/ijwh.s104160.

30. Michie S, van Stralen MM, West R. The behaviour change wheel: a new method for characterising and designing behaviour change interventions. *Implement Sci* 2011;6:42 doi: 10.1186/1748-5908-6-42.

31. Ostebo MT, Cogburn MD, Mandani AS. The silencing of political context in health research in Ethiopia: why it should be a concern. *Health Policy Plan* 2018;33(2):258-70.

32. Bohren MA, Hunter EC, Munthe-Kaas HM, et al. Facilitators and barriers to facility-based delivery in low- and middle-income countries: a qualitative evidence synthesis. *Reprod Health* 2014;11(1):71 doi: 10.1186/1742-4755-11-71.

33. Godlonton S, Okeke EN. Does a ban on informal health providers save lives? Evidence from Malawi. *J dev Econ* 2016;118:112-32 doi: 10.1016/j.jdeveco.2015.09.001.

34. Turinawe EB, Rwemisisi JT, Musinguzi LK, et al. Traditional birth attendants (TBAs) as potential agents in promoting male involvement in maternity preparedness: insights from a rural community in Uganda. *Reprod Health* 2016;13:24 doi: 10.1186/s12978-016-0147-7.

35. Rudrum S. Traditional birth attendants in rural Northern Uganda: policy, practice, and ethics. *Health Care Women Int* 2016;37(2):250-69 doi: 10.1080/07399332.2015.1020539.

36. Sarelin A. Modernisation of maternity care in Malawi. *Nord J Hum Rights* 2014;32(4):331-51 doi: 10.1080/18918131.2014.957463[|.

For peer review only - http://bmjopen.bmj.com/site/about/guidelines.xhtml

37. Banda E. Stakeholders' perceptions of the changing role of traditional birth attendants in the rural areas of Central West Zone, Malawi: a mixed methods study. PhD thesis, University of the Witwatersrand, 2013.

38. Rishworth A, Dixon J, Luginaah I, et al. "I was on the way to the hospital but delivered in the bush": maternal health in Ghana's Upper West Region in the context of a traditional birth attendants' ban. *Soc Sci Med* 2016;148:8-17 doi: 10.1016/j.socscimed.2015.11.018.

39. Cheelo C, Nzala S, Zulu JM. Banning traditional birth attendants from conducting deliveries: experiences and effects of the ban in a rural district of Kazungula in Zambia. *BMC Pregnancy Childbirth* 2016;16(1):323 doi: 10.1186/s12884-016-1111-9.

40. Eggermont M. The choice of child delivery is a European human right. *Eur J Health Law* 2012;19(3):257-69.

41. Stromerova Z. Midwifery in East Europe: challenges and solutions. *Midwifery Matters* 2012(133):4-6.

42. Melberg A, Diallo AH, Ruano AL, et al. Reflections on the unintended consequences of the promotion of institutional pregnancy and birth care in Burkina Faso. *PLoS One* 2016;11(6).

43. Hornik RC. Exposure: Theory and evidence about all the ways it matters. *Soc Mark Q* 2002;8(3):31-37 doi: 10.1080/15245000214135.

44. Murray J, Remes P, Ilboudo R, et al. The saturation+ approach to behavior change: case study of a child survival radio campaign in Burkina Faso. *Glob Health Sci Pract* 2015;3(4):544-56 doi: 10.9745/ghsp-d-15-00049.

45. Aubel J, Toure I, Diagne M. Senegalese grandmothers promote improved maternal and child nutrition practices: the guardians of tradition are not averse to change. *Soc Sci Med* 2004;59(5):945-59 doi: 10.1016/j.socscimed.2003.11.044.

46. Dumbaugh M, Tawiah-Agyemang C, Manu A, et al. Perceptions of, attitudes towards and barriers to male involvement in newborn care in rural Ghana, West Africa: a qualitative analysis. *BMC pregnancy and childbirth* 2014;14:269 doi: 10.1186/1471-2393-14-269.

47. Iganus R, Hill Z, Manzi F, et al. Roles and responsibilities in newborn care in four African sites. *Trop Med Int Health* 2015;20(10):1258-64 doi: 10.1111/tmi.12550.

48. Polit DF, Beck CT. Generalization in quantitative and qualitative research: myths and strategies. *Int J Nurs Stud* 2010;47(11):1451-8 doi: 10.1016/j.ijnurstu.2010.06.004.

49. Finfgeld-Connett D. Generalizability and transferability of meta-synthesis research findings. *J Adv Nurs* 2010;66(2):246-54 doi: 10.1111/j.1365-2648.2009.05250.x.

For peer review only

For peer review only

For peer review only - http://bmjopen.bmj.com/site/about/guidelines.xhtml

For peer review only

For peer review only - http://bmjopen.bmj.com/site/about/guidelines.xhtml

Reporting checklist for qualitative study.

Based on the SRQR guidelines.

Instructions to authors

Complete this checklist by entering the page numbers from your manuscript where readers will find each of the items listed below.

Your article may not currently address all the items on the checklist. Please modify your text to include the missing information. If you are certain that an item does not apply, please write "n/a" and provide a short explanation.

Upload your completed checklist as an extra file when you submit to a journal.

In your methods section, say that you used the SRQR reporting guidelines, and cite them as:

O'Brien BC, Harris IB, Beckman TJ, Reed DA, Cook DA. Standards for reporting qualitative research: a synthesis of recommendations. Acad Med. 2014;89(9):1245-1251.

For peer review only

		Reporting Item	Page Number
	#1	Concise description of the nature and topic of the study identifying the study as qualitative or indicating the approach (e.g. ethnography, grounded theory) or data collection methods (e.g. interview, focus group) is recommended	4
	#2	Summary of the key elements of the study using the abstract format of the intended publication; typically includes background, purpose, methods, results and conclusions	2
Problem formulation	#3	Description and signifcance of the problem / phenomenon studied: review of relevant theory and empirical work; problem statement	3
Purpose or research question	#4	Purpose of the study and specific objectives or questions	3
Qualitative approach and research paradigm	#5	Qualitative approach (e.g. ethnography, grounded theory, case study, phenomenolgy, narrative research) and	3

		guiding theory if appropriate; identifying the research paradigm (e.g. postpositivist, constructivist / interpretivist) is also recommended; rationale. The rationale should briefly discuss the justification for choosing that theory, approach, method or technique rather than other options available; the assumptions and limitations implicit in those choices and how those choices influence study conclusions and transferability. As appropriate the rationale for several items might be discussed together.	
Researcher characteristics and reflexivity	#6	Researchers' characteristics that may influence the research, including personal attributes, qualifications / experience, relationship with participants, assumptions and / or presuppositions; potential or actual interaction between researchers' characteristics and the research questions, approach, methods, results and / or transferability	6
Context	#7	Setting / site and salient contextual factors; rationale	4
Sampling strategy	#8	How and why research participants, documents, or events were selected; criteria for deciding when no further sampling was necessary (e.g. sampling saturation); rationale	4
Ethical issues pertaining to human subjects	#9	Documentation of approval by an appropriate ethics review board and participant consent, or explanation for lack thereof; other confidentiality and data security issues	6
Data collection methods	#10	Types of data collected; details of data collection procedures including (as appropriate) start and stop dates of data collection and analysis, iterative process, triangulation of sources / methods, and modification of procedures in response to evolving study findings; rationale	4-6
Data collection instruments and technologies	#11	Description of instruments (e.g. interview guides, questionnaires) and devices (e.g. audio recorders) used for data collection; if / how the instruments(s) changed over the course of the study	5
Units of study	#12	Number and relevant characteristics of participants, documents, or events included in the study; level of	5

For peer review only

		participation (could be reported in results)	
Data processing	#13	Methods for processing data prior to and during analysis, including transcription, data entry, data management and security, verification of data integrity, data coding, and anonymisation / deidentification of excerpts	6
Data analysis	#14	Process by which inferences, themes, etc. were identified and developed, including the researchers involved in data analysis; usually references a specific paradigm or approach; rationale	6
Techniques to enhance trustworthiness	#15	Techniques to enhance trustworthiness and credibility of data analysis (e.g. member checking, audit trail, triangulation); rationale	6
Syntheses and interpretation	#16	Main findings (e.g. interpretations, inferences, and themes); might include development of a theory or model, or integration with prior research or theory	7-10
Links to empirical data	#17	Evidence (e.g. quotes, field notes, text excerpts, photographs) to substantiate analytic findings	7-10
Intergration with prior work, implications, transferability and contribution(s) to the field	#18	Short summary of main findings; explanation of how findings and conclusions connect to, support, elaborate on, or challenge conclusions of earlier scholarship; discussion of scope of application / generalizability; identification of unique contributions(s) to scholarship in a discipline or field	11-12
Limitations	#19	Trustworthiness and limitations of findings	12
Conflicts of interest	#20	Potential sources of influence of perceived influence on study conduct and conclusions; how these were managed	12
Funding	#21	Sources of funding and other support; role of funders in data collection, interpretation and reporting	1

The SRQR checklist is distributed with permission of Wolters Kluwer © 2014 by the Association of American Medical Colleges. This checklist can be completed online using https://www.goodreports.org/, a tool made by the EQUATOR Network in collaboration with Penelope.ai

For peer review only

BMJ Open

“People have started to deliver in the facility these days”: A qualitative exploration of factors affecting facility delivery in Ethiopia

Journal:	*BMJ Open*
Manuscript ID	bmjopen-2018-025516.R2
Article Type:	Research
Date Submitted by the Author:	07-Mar-2019
Complete List of Authors:	Hill, Z; University College London, Institute for Global Health<br>Amare, Yared; Consultancy for Social Development<br>Scheelbeek, Pauline; London School of Hygiene and Tropical Medicine<br>Schellenberg, Joanna; London School of Hygiene & Tropical Medicine, Dept of Infectious Disease Epidemiology
**Primary Subject Heading**:	Public health
Secondary Subject Heading:	Global health, Health services research, Qualitative research
Keywords:	facility delivery, qualitative, ethiopia, africa, community health workers

SCHOLARONE™
Manuscripts

"*People have started to deliver in the facility these days*": A qualitative exploration of factors affecting facility delivery in Ethiopia

Dr Zelee Hill (corresponding author), University College London, 30 Guilford Street, London WC1N 1EH, UK. E mail: z.hill@ucl.ac.uk

Dr Yared Amare, Consultancy for Social Development, PO Box 70196, Addis Ababa, Ethiopia. yaredamare@yahoo.com

Dr Pauline Scheelbeek, London School of Hygiene and Tropical Medicine, Keppel Street, London, WC1E 7HT, UK. pauline.scheelbeek@lshtm.ac.uk

Professor Joanna Schellenberg, London School of Hygiene and Tropical Medicine, Keppel Street, London, WC1E 7HT, UK. joanna.schellenberg@lshtm.ac.uk

For peer review only

Abstract

Objectives: To understand the recent rise in facility deliveries in Ethiopia
Design: A qualitative study
Setting: Four rural communities in two regions of Ethiopia
Participants: 12 narrative, 12 in-depth interviews and four focus group discussions with recently delivered women; and four focus group discussions with each of grandmothers, fathers and community health workers.
Results: We found that several interwoven factors led to the increase in facility deliveries, and that respondents reported that the importance of these factors varied over time. The initial catalysts were a saturation of messages around facility delivery, improved accessibility of facilities, the prohibition of TBAs, and elders having less influence on deciding the place of delivery. Once women started to deliver in facilities, the drivers of the behavior changed as women had positive experiences. As more women began delivering in facilities, families shared positive experiences of the facilities, leading to others deciding to deliver in a facility.
Conclusion: Our findings highlight the need to employ strategies that act at multiple levels, and that both push and pull families to health facilities.

Key words: Facility delivery; Ethiopia, Qualitative

Word count: 4355

Article Summary: Strengths and limitations:

- The use of qualitative methods to understand a complex issue
- Use of multiple study sites, purposive sampling to saturation, reflexivity, triangulation of methods and respondent groups, and within and cross case analysis
- The study sites were all reasonably accessible, within half an hour walk from a motorable road, and had reasonably functioning Health Extension Worker systems. The study may thus underestimate accessibility issues, and the mechanism related to message saturation is unlikely to be triggered where Health Extension Workers function sub-optimally.
- There is the potential for social desirability bias given the pressure for women to deliver in a health facility, and because study mothers were mainly identified by Health Extension Workers or members of the Health Development Army.

Funding statement: This work was supported by the Bill and Melinda Gates Foundation as part of the IDEAS study

Competing interests: The authors have no competing interests

Data availability statement: Unpublished data from this study are not available. Data anonymity is not obtainable given the depth of the narrative qualitative transcripts, even when these have been anonymised.

Author contributions: Zelee Hill (ZH) and Joanna Schellenberg (JS) conceived the study. ZH, Yared Amare (YA) and Pauline Scheelbeek (PS) designed the data collection tools and strategy, YA and PS oversaw data collection, ZH, AY and PS conducted the analysis. ZH wrote the first draft of the manuscript and all authors contributed to the re-drafting and revision of the manuscript.

For peer review only

Introduction

Sub-Saharan Africa accounts for an estimated 66% of all maternal deaths.[1] Despite recent declines, mortality rates are still unacceptably high. Most deaths are preventable and occur around the time of delivery. Although it is essential to address the broad determinants of maternal mortality, such as female education and social status,[2] having a skilled attendant at delivery is nevertheless considered to be the most critical intervention.[3 4]

Ethiopia had one of the lowest levels of facility delivery coverage in the world, with Demographic and Health Survey (DHS) data showing coverage of 5% in 2000, 6% in 2005, and 11% in 2011 (three year recall).[5] This low coverage persisted despite government efforts to increase the accessibility and availability of services.[6 7] Efforts included the introduction of the Health Extension Program in 2003, where two female Health Extension Workers (HEW), stationed at newly constructed rural health posts, and paid around $100 a month, were trained for one year to serve around 5,000 people. They provide health promotion, disease prevention and treatment, and work both at the health post and in the community.[8-11] Their role includes providing care to women through pregnancy, birth and post-natally including providing ANC and promoting birth preparedness and complication readiness. [12] The program had little impact on the coverage of skilled delivery, even when Health Extension Workers were trained to conduct deliveries in the health post,[7 12] and studies have found persistent and multiple barriers to change.[13-20] HEWs no longer provide delivery services but rather assist delivering women in reaching health centers staffed by skilled birth attendants. [21]

By 2016 the DHS, using the same methodology to measure coverage as in previous surveys, found that coverage increased to 33% (3 year recall, with a regional range of 18-97%), a 200% increase from 2011.[22] The Ethiopian government attributes the increase largely to the work of the Health Development Army (HDA).[23-25] The HDA, created in 2012, is a network of all women in rural areas, organized into development groups of 30 women (1-30 networks), who are further clustered into groups of 6 (1-5 networks).[20 26-28] Groups select a leader who is then trained and supervised by the HEW. The HDA leaders help members adopt practices promoted by the HEW, hold participatory learning and action meetings, link pregnant women with care providers, hold monthly meetings for pregnant women, mobilize communities to contribute resources to make facilities mother friendly, and facilitate the use of either traditional or modern ambulances.[11 20 23 27]

Around the same time as the creation of the HDA, there were other policy changes that could have influenced facility delivery rates. These include the prohibition of the use of Traditional Birth Attendants (TBAs) for delivery,[20 26] and the provision of a four-wheel drive ambulance to transport women to facilities for delivery to every rural district.[29] In addition the number of health centers, staffed with two midwives, increased and the road infrastructure was also improved.[26 30] In this paper we use qualitative data to explore the reasons for the increase in facility deliveries in four study sites, we used a phenomenological approach as we were interested in understanding lived experiences.

For peer review only - http://bmjopen.bmj.com/site/about/guidelines.xhtml

Methods:

Study setting selection and characteristics: Data were collected between March and May 2015, from two wards (*kebeles),* the smallest unit of local government, in the Southern Nations, Nationalities and Peoples region (SNNP) and two in Amhara region. Amhara has shown an increase in facility deliveries from 11% (2011) to 35% (2016), and SNNP region from 7% to 33%.[5]

Data were collected from areas where 'The Last Ten Kilometers' (L10K) programme was active in supporting the Health Extension Program. *Kebeles* were selected from a list, provided by L10K project staff, of *kebeles* considered to have a reasonably functioning HEW system, that is that they had HEWs in place that were considered to be active and working well. Other selection criteria were that the *kebeles* were seen as typical of the district (*woreda*) with no unusual characteristics such as having a large hospital or a large industry close by, and were less than half an hours walk from a motorable road so that the study team could feasibly access them. We have labeled these *kebele* "A-D" to maintain anonymity. Table 1 shows the characteristics of the selected *kebele*s, all of which had a subsistence farming based economy. Although the study sites were all a short walk from a motorable road, access to public transport was very limited.

Table 1. Characteristics of Study *kebele*

Region	*Kebele*	Ethnicity	Predominant religion	Access to health centers	Terrain
Amhara	*Kebele* A	Amhara	Orthodox Christian	Moderate	Hilly
	Kebele B	Amhara	Orthodox Christian	High	Hilly
SNNPR	*Kebele* C	Gamo/ Wolaita	Protestant/ Orthodox Christian	High	Predominantly flat with some hilly parts
	Kebele D	Silte	Muslim	Moderate	Flat

Data collection: Data were collected as part of a study to understand how HEWs influence maternal and newborn care behaviors, of which facility delivery was one. Four trained interviewers collected data in the local language using pre-tested semi structured guides developed by the authors. When needed translators were used. The content of the guides was informed by a theoretical framework, which identified pathways through which HEWs could influence behaviors by modifying families capabilities, opportunities and motivation.[31] Data were collected from mothers, grandmothers, fathers, HEWs and HDA using narrative interviews, in-depth interviews, and focus group discussions. All community respondents had children or grandchildren under 12 months of age, with narrative mothers having children less than three months of age to facilitate recall. Using a range of both methods and respondents allowed for data triangulation and ensured we captured a range of viewpoints. Narrative interviews with mothers were used to capture personal experiences, in-depth interviews to capture perceptions of what was commonly done in the community, and focus group discussions to collect data that we felt would benefit from being discussed in a group interaction.

For peer review only - http://bmjopen.bmj.com/site/about/guidelines.xhtml

Data were collected until saturation was reached, i.e. until additional interviews provided similar information to that already obtained. Saturation was determined by frequent transcript reviews. The sample size, respondent groups, and the interview content related to facility delivery are shown in Table 2. In the FGD we employed several activity oriented exercises such as sorting and ranking and root cause analysis to encourage group interaction and participation and reduce social desirability bias, which can be a particular issue in Ethiopia.[32]

For peer review only

Table 2: Data collection method, sample size and content related to facility delivery

Method	Sample	Interview content related to facility delivery
Narrative interviews with recent mothers	12	• Labor and delivery story<br>• Perceived knowledge and skills related to pregnancy and newborn care<br>• Description of contacts during pregnancy and delivery with health workers, HDAs and HEWs<br>• Information received on where to deliver, source of the information, reaction to the information, and the impact of the information on decision making
In-depth interviews with recent mothers	13	• Perceptions of where most people deliver, and community views of those who deliver at home and those who deliver in a facility<br>• Views on HEW/HDA work and their suitability<br>• Description of contacts during pregnancy and delivery with health workers, HDAs and HEWs<br>• Information received on where to deliver, source of the information and reaction to the information<br>• Most significant maternal and newborn health changes in the community in the last 2 years, and why things changed
FGD with recent mothers	4	• Pile sort of behaviors practiced/not practiced, important/not important, and that are promoted/not promoted by HEWs/HDAs<br>• Community views of those who deliver at home and those who deliver in a facility<br>• Most significant maternal and newborn health changes in the last 2 years, and why things changed<br>• Reaction to statements that HEWs/HDAs work does not bring change, and that people dislike HEWs telling them where to deliver
FGD with grand-mothers	4	• Reaction to a picture of a facility delivery<br>• Most significant maternal and newborn health changes in the last 2 years and why things changed<br>• Reaction to statements about grandmothers supporting traditional practices, and that mothers do not listen to grandmother advice
FGDs with fathers	4	• Reaction to a picture of a facility delivery<br>• Fathers role in deciding place of delivery<br>• Response to a scenario where a family does not follow HEW advice<br>• Views on HEW/HDA work and their suitability<br>• Most significant maternal and newborn health change in the last 2 years and why things changed<br>• Reaction to statements that mothers/fathers make decisions about delivery, and that people dislike HEWs telling them where to deliver
FGD with HEW and HDA	4	• Pile sort of behaviors practiced/not practiced, important/not important, and that are promoted/not promoted by HEWs/HDAs<br>• Most significant changes in the community, and in their work, related to maternal and newborn health in the last 2 years and why things changed<br>• Successes and challenges they faced in encouraging behavior

For peer review only

		change<br>• Reaction to statements that mothers get punished if they do not follow advice, and that mothers prefer advice of family members

Mothers, grandmothers and fathers, from different households, were identified by the HEW/HDA, or through snowball sampling from the community respondents – with the first method providing the majority of respondents. Eligibility criteria were that the family had received at least one visit by an HEW or HDA. Mothers were selected to ensure diversity in age, educational level, parity, sex of newborn and socio-economic status. Grandmothers could be paternal or maternal – as long as they had been dependent on which was closest to the family. We also aimed to get diversity in place of delivery, but located few women who admitted they delivered at home. All of the HDAs in the study *kebeles* were invited for the HDA FGDs. As there were only 2 HEWs per *kebele* HEW FGDs included HEWs from neighboring *kebeles.* Interviewers approached potential respondents in their home, or at the health post. Three respondents refused, as they were too busy. Interviews lasted from 1 to 2 hours and took place in respondents' houses, or the health post for the HEW. FGDs were conducted with 3-7 *respondents* in neutral locations and lasted from 1.5 to 2.5 hours. HEWs and HDAs were not present during any of the interviews or FGDs with community members.

Interviews and FGDs were audio-recorded and fully transcribed by the data collectors in English as soon as possible. Data collectors met regularly during fieldwork to discuss emerging themes and to receive feedback from the senior researchers. Ethical approval was granted by the Ministry of Science and Technology in Ethiopia and the London School of Hygiene and Tropical Medicine in the UK. Written informed consent was obtained from all respondents. On entering a household the interviewer introduced themselves and the project to key people, and gave the head of household a project leaflet. They explained who they wanted to interview read aloud a study information sheet to them in a quiet place. For FGDs the information was read aloud to all FGD respondents. The interviewers checked respondents' comprehension, rephrased if necessary and gave the respondents an opportunity to ask questions. If the respondent agreed to be interviewed the interviewer read the consent form out loud and asked the respondent to sign to show that they were willing to be interviewed, understood the study, were happy for their words to be written down and recorded, were happy for their quotes to be used, and for the information collected to be transferred to London. The interviewers also signed each form.

Respondent and public involvement: Respondents were not directly involved in the design of the study, however the interview guides were iterative and were modified as the research progressed based on reported experiences and perceptions. Some respondents were recruited through snowball sampling, i.e. where respondents suggested others they knew who were eligible for interview.

Data analysis: Analysis began during data collection through regular team meetings and reflection. A formal analysis session was held with the data collectors in the middle and at the end of data collection, this included discussion of how our characteristics could have influenced how data were collected and interpreted. Once data were collected all transcripts were read several times to ensure familiarity with the data, to begin to identify notable constructs and to see the data as a whole. A deductive coding template was developed in Nvivo based on the theoretical framework that guided the interview content. Interviews and focus groups were then coded inductively within these broad themes. Coding was done by identifying the underlying meaning of each section of text and how it was different or similar

For peer review only

to others section. Codes that contained similar concepts were then put into larger themes. Themes and codes were modified by looking for patterns, links and contradictions within themes. Data credibility was checked by triangulating data between respondent groups and between data collection methods. Data analysis was done by three of the senior researchers, who discussed their coding regularly to enhance conceptual thinking and to increase coding rigor. Reflective notes were kept throughout the process.

For peer review only

Results

Table 3 shows the characteristics of the narrative and IDI respondents. Respondents had a range of ages, education levels, parities, and religion. We did not achieve the planned diversity in place of delivery, as 19/25 of the narrative/IDI women had delivered in a facility. This is possibly because families were reluctant to admit to home deliveries, and because facility delivery rates may have been high in the study area because the sites were relatively accessible, within walking distance of a motorable road, and had functioning HEW systems. In addition HEW/HDAs assisted in identifying respondents, and may have favored those who delivered in a facility.

The FGD participant mothers were varied in age (range 19-35 years of age), parity (range 1-7 children), education (none-secondary level), and ethnicity. The FGD participant fathers were older (range 28-45 years of age) and the FGD participant grandmothers were less educated with almost all being uneducated. Grandmothers were predominantly, but not exclusively, paternal.

Table 3: Sample characteristics (narrative and mother IDIs)

Characteristic	Frequency (n=25)
**Age**	
≤ 24	10
25-34	10
≥35	5
**Education**	
None	10
Primary	12
Secondary and above	3
**Religion**	
Islamic	8
Christian	17
**Parity**	
1	7
2-3	7
≥4	11
**Place of last delivery**	
Home	6
Facility	19

All respondent groups reported that the increase in facility delivery was recent, and that previous attempts to encourage facility delivery had limited success: '*People have started to deliver in the facility these days……. They* [families] *used to give us lots of excuses like, let the cattle return back home, let the sun start setting, and let's wait for this and that; believing that the mother would deliver in the meantime….so that used to be very problematic'* [HEW FGD, *kebele* A Amhara]. At the time of data collection, delivering in a health center was reported as the usual practice in all study communities, and respondents reported that: *'everyone knows what to do'… 'no one delivers at home'*.

For peer review only - http://bmjopen.bmj.com/site/about/guidelines.xhtml

Respondents reported that the factors that influenced facility delivery changed over time and consisted of push and pull factors. We identified four themes around the initial uptake of facility delivery: saturation of messages around facility delivery, improved accessibility of facilities, the prohibition of TBAs, and elders having less influence on deciding the place of delivery. Themes around the drivers of facility delivery once uptake had begun were around families having positive experiences of facilities, seeing the worth of a facility delivery, and sharing their positive experiences with others.

Saturation of messages and contacts: All mothers interviewed reported receiving information on the importance of facility delivery, and all respondent groups knew that it was being strongly promoted at: *'every opportunity'.* In the majority of cases mothers had received information from at least two sources, and at several time points: '[HDA] *tells me about it repeatedly and forcefully'* [Mother IDI, *kebele* D SNNPR]. Information was mainly given at the health post, at home, during ANC, and at community meetings. The main sources of information were the HEWs and HDAs. The resultant high awareness levels was reported as a reason for the increase in facility delivery rates:

> *Interviewer: Why didn't you go* [to the facility] *at that time* [for previous deliveries]*?*
> *Respondent: Because there was no one who educates you like this at that time. Nobody advised us to deliver in the health center…… My knowledge was not as strong at that time* [Mother IDI, *kebele* D SNNPR]

A theme that emerged from all respondent groups, related to how the information on facility delivery was received, was around community trust in health workers and HEWs. Trust in HEWs arose from a view that HEWs were knowledgeable because of their training and were higher status than community members: *'They* [HEW] *are better than us; they teach us what they have learned. She* [HEW] *went there* [training] *so that she could bring us some good education, we don't believe she teaches us harmful advice'* [Mother FGD, *kebele* D SNNPR]. The theme around trust in HEWs was contrasted by views of the had leaders who were less trusted as they were viewed as people who transferred messages rather than being knowledgeable in their own right. But, HDA leaders played a key role in ensuring the penetration of messages, and by informing HEWs about pregnant women: *'The leaders of this group* [1-5 HDA group] *follow how many of them are pregnant …. The leaders know everything about their group…… And when labor starts, the leader will inform the HEWs'* [Mother IDI, *kebele* B Amhara].

Improved accessibility: Knowledge of the ambulance service was universal across respondent groups. In some sites families were given the ambulance number during pregnancy. In other sites families called the HEW at the start of labor, and the HEWs then called the ambulance. The presence of the ambulance was reported as facilitating facility delivery by all groups:

> *'If ambulance service had not started functioning in the kebele, the mother surely gives birth at home'* [Mother FGD, *kebele* A Amhara]
>
> *'This time there is no one who delivered at home, it was in our fathers' time, now there is ambulance which take the mother to the health center, so all women deliver there'* [Mother FGD, *kebele* A Amhara]

A theme among fathers was the role that the increase in the number of health centers, free delivery care and the construction of roads had impacted on facility delivery uptake: *'Formerly people think there is a payment for delivery, like they pay for treatment but there*

For peer review only - http://bmjopen.bmj.com/site/about/guidelines.xhtml

is no such things … there were a lot of people who deliver at home thinking it [facility delivery] *needs money… even if she* [mother] *asked to go the husband didn't want, thinking he will be asked for money'* [Father FGD, *kebele* D SNNPR].

Despite the reported importance of ambulances, several families interviewed described a problem accessing the service. This was most frequently because the ambulance was busy, could not come because of heavy rain, had no fuel, or took too long to come: *'I asked* [HEW] *to call an ambulance, but there was no ambulance so we were told to use public transport'* [Grandmother FGD, *kebele* B Amhara]. In the narrative interviews over half of the women (6/9) who called an ambulance had a problem accessing the service, of these 3 delivered at home or with the HEW, 1 delivered in the health center but waited a long time for the ambulance and the other 2 took public transport.

The four study sites were reasonably accessible and respondents talked of villages where health facility delivery was still very difficult because of accessibility issues: '*Not all villages are accessible, those who live in X village, they are not able to deliver in health facility. But those who live in nearer villages….. It is a must to deliver in health facility since they are close'* [Mother IDI, *kebele* A Amhara].

Prohibition of TBAs: In all sites the use of traditional birth attendants was reported as forbidden, with a threat of sanctions for those who conducted or had a home delivery:

> *During pregnancy, they* [HEW] *told me not to deliver at home. They said "if you deliver at home, you will be punished"….. 500 birr* [$22] *if I deliver at home* [Mother IDI, *kebele* C SNNPR]

> *….birth attendants are not willing to assist due to fear of punishment….. the women herself will pay 2000 birr* [$88] *and the birth attendant will pay 1000 birr* [$44] [Mother IDI, *kebele* B Amhara]

Decisions about sanctions were made at community level through the 1-5 or 1-30 HDA networks: *'The community decided a 'Sera'* [customary law] *that if a mother delivers at home she will be fined 500 birr* [$22]*'* [Narrative interview, *kebele* B Amhara]. The reported sanctions were varied, with the fines ranging from 200-2000 birr [$9-88].

The possibility of being sanctioned for delivering at home was a key theme relating to the initial uptake of messages around facility deliveries among all respondent groups:

> Interviewer: *What do you think brought this change*?
> Respondent:…. *the fear of the punishment, I don't think most of the community understood the benefit of delivering in the facility…. didn't give due attention to the lessons….. it is after we are told we will be punished* [Mother IDI, *kebele* C SNNPR]

> *Respondent 2: There is 1 to 5* [HDA group]*, and one watch over the other, and there is also punishment; if the mother deliver at home she will be fined with 500 birr* [$22].
> *Respondent 3: There is a law they are fined …. after that people start saying hurry up please she is going to deliver* [laughing] [Father FGD, *kebele* C SNNPR]

For peer review only

Respondents, including former TBAs, reported that the sanctions were justified and beneficial as times were changing. HEWs were aware of them, and at times encouraged their use:

> *Respondent: When a mother delivers at home and if the baby dies….the government will prosecute her for that….. We use such threats.*
> *Interviewer: Who tells them such threats?*
> *Respondent: We call the HDA leaders* [1 to 30] *and then tell them that such threats may work, and then they go and tell the mothers* [HEW FGD, *kebele* C SNNPR]

In only one site were there reports that sanctions had been used in practice, and the respondents that we interviewed who had delivered at home reported that they had been excused the sanctions: '[HEW said] *If you were another person I will take you to jail but you face a lot of problem that's why I left you'* [IDI mother, *kebele* D SNNPR]. Although the respondents who delivered at home reported that sanctions were not applied, they did report that the HEWs were angry with them and, in a few cases, denied them services: *'She* [HEW] *suspected that I hid and delivered at home….. and was very angry. Because she was angry then, she did not tell me things…..there was no mention of how I should be bathing the baby and the like'* [Narrative woman, *kebele* B Amhara].

Power shift: Grandmothers had little influence on place of delivery, and respondents in all groups used words like: *'we are in a different time'* and *'time has changed'.* This lack of influence was attributed to mothers being modern because of the education given by the HEW, were thus more knowledgeable than their elders, and were consequently able to challenge their advice: *Today's mothers are young and modern. They easily accept new ideas……. they wouldn't like to do the traditional practice ….. since they have received the new education* [Narrative mother, *kebele* C SNNPR]. Husbands were viewed as having the ultimate decision making power in the household and generally supported facility delivery. This support from husbands put mothers in a stronger position if they faced opposition from their elders. We found very few grandmothers who reported that they were resistant to the change in delivery location.

Positive experiences: The main theme around how the drivers of facility delivery changed over time was around the influence of families having and sharing positive experiences of facility delivery:

> *I felt very happy* [to deliver in the facility]…. *The doctors give morale, they said take it easy, be strong and the like'* [Mother IDI, *kebele* D SNNPR].

> *But now, it is not that they* [families] *are afraid of the punishment, they have started saying that they are going because they want to get care from the health professionals…. They have started saying that the physicians do all they can and help them deliver* [HDA FGD, *kebele* D SNNPR]

> *Formerly they feared the penalty, but now those who delivered there* [at health center] *talk about good thing of delivering there* [Mother IDI, *kebele* C SNNPR]

All respondents in all groups reported that facility delivery was safer and reduced deaths. The provision of an injection to stop bleeding was the most frequently mentioned benefit along with getting a vaccination for the baby, removal of dirt from the abdomen/stomach, the baby being cared for and not left alone, the facility being hygienic, delivery being less

For peer review only - http://bmjopen.bmj.com/site/about/guidelines.xhtml

painful and more predictable, and that the facility could deal with problems such as the baby being in the wrong position, the placenta getting stuck, or the baby being born weak:

> *I know that I will not be hurt if I deliver in the health center….. they will inject me something which stopped the excessive bleeding; I know that they will assist me if I will have any complications* [Mother IDI, *kebele* C SNNPR]

> *Here* [home] *there is only suffering until the delivery nothing else, and we are delivering in the facility in a very relaxed way…… they* [health workers] *measure and tell us how much time is left……but here we don't know anything, we are just laboring and waiting until we deliver or die* [Mother IDI, *kebele* D SNNPR]

All respondent groups reported that at the facility mothers received food, drink and sometimes a cloth for the baby: *'Everything is perfect, even porridge and gruel is prepared and served to mothers in the health center. It is really good…….. Even the person who accompanied them is invited'* [Grandmother FGD, *kebele* B Amhara]. A theme related to grandmothers was that becasue they were allowed in the delivery room they had less fears about what occurred during a facility delivery: *'We* [health workers] *never used to allow anybody inside the delivery … as they* [family members] *start to see; they started saying 'we were afraid that you would insert materials inside her' …. they see that things are good'* [HEW FGD, *kebele* A Amhara].

Not only did respondents report benefits and good experiences of delivering at the facility, they often shared their experience with their friends and neighbors, and several mothers reported that their friends and neighbors had influenced their delivery location:

> *I heard from other people, I heard that it is good to deliver in the health center, so I was planning to deliver there…. one of my neighbors delivered in the health facility, and she told me it is good…. She told me the mother will be very clean, she will not have bleeding….. health professionals would help the mother and save her life*. [Mother narrative, *kebele* C SNNPR]

> *Respondent 1: Now, the unwilling ones also went there because she saw when others do.*
> *Respondent 2: Yes, formerly, they did not want to be exposed. They said that, Saint Mary will do what she wants. But now, they see the benefit. And learn one from the other'* [HDA FGD, *kebele* B Amhara]

For peer review only - http://bmjopen.bmj.com/site/about/guidelines.xhtml

Discussion

A systematic review of qualitative studies exploring facility delivery classified the findings based on the quality and coherence of studies.[33] Barriers to facility delivery in which there was high confidence were: cultural barriers, such as perceptions of birth as a natural event; decision making barriers, including the role of elder women; proximity, access and cost barriers; a reliance on TBAs; and barriers related to perceived poor quality of care and mistreatment by health workers. High confidence facilitators were valuing facilities for complications and perceiving them as providing high quality of care. Previous birth experiences were both a barrier and a facilitator. Previous studies specific to Ethiopia identified similar barriers and.[13-20] In our study none of these barriers were reported, with the exception of accessibility issues for more remote villages. Our findings suggest that these barriers were overcome through a combination of saturation of messages around facility delivery from trusted sources, reduction in access issues, the prohibition of TBAs, power shifts away from grandmothers and positive experiences. The focus of this paper on what has driven the change process adds new insights to the literature, which to date has focused on barriers and facilitators to uptake rather than mechanisms of change. It is widely recognized that comprehensive efforts, at multiple levels, are required to successfully increase facility delivery rates,[33] this is what has occurred in the study sites. Previous interventions In Ethiopia, that have focused on access barriers at one level have not been successful.[30]

Respondents reported that the drivers of behavior change in our study sites varied over time. One of the initial catalysts was the prohibition of TBAs. TBAs have been prohibited in several other African countries, but the policy has often encountered problems such as the ban being ineffectual due to enforcement issues, TBAs continuing their work underground, accessibility remaining a key barrier, and poor quality facilities limiting the effects.[34-40] Our data suggest that, in our study sites, the ban has been effective. This could be because the ban was coupled with increased awareness of and access to alternative options, that were viewed positively, and because the HDA model allowing pregnant women to be identified and followed. We were unable to locate details of the TBA ban, but our data suggest that the specifics of the ban, in relation to whether and how it was implemented, were determined at local levels. There may be considerable variation in implementation and impact in other Ethiopian settings.

Prohibiting home births is controversial, and it has been argued that it infringes on personal choice and autonomy.[36 37] This is exemplified in a ruling in the European Court of Human Rights that regulations which make home births difficult to obtain violate the right to a private life.[41 42] On the other hand it has been argued that the restrictions implemented in several African countries are made in the interest of public health and are thus justified. Whatever view is taken, an important consideration is whether such bans and sanctions result in families hiding home deliveries, and subsequently reducing their care seeking. For example, in Burkina Faso sanctions and verbal abuse for those who did not attend services resulted in these families being too fearful to access services when they needed them.[43]

In our study as families began to experience facility deliveries, the driver changed from push factors to the pull of a desire for facility deliveries. Positive experiences at the facility changed perceptions, and neighbors shared their experiences. If families continue to have and share positive experiences the increase is likely to be sustained.

For peer review only - http://bmjopen.bmj.com/site/about/guidelines.xhtml

Saturation of messages was a key driver in increasing awareness and uptake of facility deliveries. The importance of achieving saturation of messages is often overlooked within public health behavior change, and achieving high exposure has received less attention than the development of high quality messages, yet high exposure appears to be equally important for success.[44 45] The HEW and HDA model, which was functional in our study sites, facilitated high exposure as pregnant women could be identified and followed.

We found that elders had lost their decision making power, because their sons and daughters, due to information from the HEW, were now perceived as being more knowledgeable. There is often an assumption that elders are resistant to change, but we found, as others have,[46] that their views can change rapidly in some circumstances.

We found that different respondent groups highlighted different reasons for change. Efforts to make facilities friendly such as allowing family in the delivery room were particularly appreciated by grandmothers while husbands, who have been identified as making financial decisions regarding delivery location in other studies,[47 48] were the only group to report on the impact of reduced costs, improved roads and a greater number of health centers. Collecting data from multiple groups is important both for the design and evaluation of interventions.

Data quality and study limitations: We took several steps to maximize data quality, and took measures to improve the transferability of our findings including: using multiple study sites, purposive sampling to saturation, reflexivity, triangulation of methods and respondent groups and within and cross case analysis.[49 50] Despite this the findings may not apply to other areas with significantly different contextual issues. For example the study sites were all reasonably accessible and had reasonably functioning HEW systems. It is likely that distance and accessibility are the main factors influencing delivery location in less accessible areas, with our respondents reporting that they knew of areas where women were unable to deliver in facilities because of distance. Studies in other settings in Ethiopia would further enhance transferability, however, the study findings suggest several issues that could be considered when exploring issues related to facility delivery coverage and the effectiveness of interventions to increase facility delivery rates in other settings.

The main limitation of our data is the potential for social desirability bias given the pressure for women to deliver in a health facility – highlighted by the difficulty we had identifying women who delivered at home. The potential for social desirability bias has been identified as particularly high in Ethiopia given a political context that may limit how freely respondents feel able to speak.[32] Although we used methods to help overcome such bias, respondents may still have been unwilling to say negative things about facility delivery, especially those identified for interview by the HEW/HDA. In addition, those respondents identified by the HEW/HDA may have been selected because of their positive attitudes and experiences. To try to reduce this we utilized snowball sampling to identify respondents, but the majority of the respondents were identified through the HEWs/HDAs. As a result, the study respondents may have had different attitudes and experiences to families that were less favored by the HEW/HDA.

Our findings highlight the need to employ strategies that act at multiple levels, and that both push and pull families to health facilities. The ability to achieve saturation and penetration of messages and to identify and follow pregnant women was a key factor in increasing facility deliveries, this is likely to have been influenced by the unique administrative and political context of Ethiopia. The increase is likely to be sustained if families' experiences of health

For peer review only - http://bmjopen.bmj.com/site/about/guidelines.xhtml

facilities continue to be positive and effort to improve the accessibility and quality of care continue; such as the provision and maintenance of ambulances, allowing family and cultural ceremonies into the delivery room, and the provision of food at the facility. Given the unique context it is difficult to transfer findings to other countries where, for example, an HDA type network may not function as well. But, we feel that the key messages of focusing interventions at multiple levels, addressing pull and push factors and ensuring saturation of messages are useful for policy makers in other settings to consider.

For peer review only

References

1. Alkema L, Chou D, Hogan D, et al. Global, regional, and national levels and trends in maternal mortality between 1990 and 2015, with scenario-based projections to 2030: a systematic analysis by the UN Maternal Mortality Estimation Inter-Agency Group. *Lancet* 2016;387(10017):462-74 doi: 10.1016/s0140-6736(15)00838-7.

2. Filippi V, Ronsmans C, Campbell OM, et al. Maternal health in poor countries: the broader context and a call for action. *Lancet* 2006;368(9546):1535-41 doi: 10.1016/s0140-6736(06)69384-7.

3. Starrs AM. Safe motherhood initiative: 20 years and counting. *Lancet* 2006;368(9542):1130-2 doi: 10.1016/s0140-6736(06)69385-9.

4. World Health Organization. Skilled attendant at birth: 2006 updates. Geneva:World Health Orghanization 2006.

5. ICF, 2012. The DHS Program STATcompiler. http://www.statcompiler.com. [Accessed December 2018].

6. Federal Ministry of Health Planning and Programming Department. Health sector strategic plan (HSDP-III), 2005/6-2009/10. Addis Ababa, Ethiopia: Federal Ministry of Health 2005.

7. Teklehaimanot HD, Teklehaimanot A. Human resource development for a community-based health extension program: a case study from Ethiopia. *Hum Resour Health* 2013;11:39 doi: 10.1186/1478-4491-11-39.

8. Banteyerga H. Ethiopia's health extension program: improving health through community involvement. *MEDICC Rev* 2011;13(3):46-9.

9. Koblinsky M, Tain F, Gaym A, et al. Responding to the maternal health care challenge: the Ethiopian health extension program. *Ethiop J Health Dev* 2010;24(Special Issue 1):105–09.

10. Legesse H, Degefie T, Hiluf M, et al. National scale-up of integrated community case management in rural Ethiopia: implementation and early lessons learned. *Ethiop Med J* 2014;52 Suppl 3:15-26

11. Maes K, Closser S, Vorel E, et al. A women's development army: narratives of community health worker investment and empowerment in rural Ethiopia. *Stud Comp Int Dev* 2015;50(4):455-78 doi: 10.1007/s12116-015-9197-z.

12. Medhanyie A, Spigt M, Kifle Y, et al. The role of health extension workers in improving utilization of maternal health services in rural areas in Ethiopia: a cross sectional study. *BMC Health Serv Res* 2012;12(1):352 doi: 10.1186/1472-6963-12-352.

13. Bedford J, Gandhi M, Admassu M, et al. 'A normal delivery takes place at home': a qualitative study of the location of childbirth in rural Ethiopia. *Matern Child Health J* 2013;17(2):230-9 doi: 10.1007/s10995-012-0965-3.

For peer review only - http://bmjopen.bmj.com/site/about/guidelines.xhtml

14. Gebrehiwot T, Goicolea I, Edin K, et al. Making pragmatic choices: women's experiences of delivery care in Northern Ethiopia. *BMC Pregnancy Childbirth* 2012;12:113 doi: 10.1186/1471-2393-12-113.

15. Gebrehiwot T, San Sebastian M, Edin K, et al. Health workers' perceptions of facilitators of and barriers to institutional delivery in Tigray, Northern Ethiopia. *BMC Pregnancy Childbirth* 2014;14:137 doi: 10.1186/1471-2393-14-137.

16. Jackson R, Tesfay FH, Godefay H, et al. Health extension workers' and mothers' attitudes to maternal health service utilization and acceptance in Adwa Woreda, Tigray Region, Ethiopia. *PLoS One* 2016;11(3):e0150747 doi: 10.1371/journal.pone.0150747.

17. Roro MA, Hassen EM, Lemma AM, et al. Why do women not deliver in health facilities: a qualitative study of the community perspectives in south central Ethiopia? *BMC Res Notes* 2014;7:556 doi: 10.1186/1756-0500-7-556.

18. Shifraw T, Berhane Y, Gulema H, et al. A qualitative study on factors that influence women's choice of delivery in health facilities in Addis Ababa, Ethiopia. *BMC Pregnancy Childbirth* 2016;16(1):307 doi: 10.1186/s12884-016-1105-7.

19. Sipsma H, Thompson J, Maurer L, et al. Preferences for home delivery in Ethiopia: provider perspectives. *Glob Public Health* 2013;8(9):1014-26 doi: 10.1080/17441692.2013.835434.

20. Zerihun A, Admassu M, Tulloch O, et al. Report on the context analysis: close-to community providers in Ethiopia, Hawassa, Ethiopia: Reach Out 2014.

21. Federal Ministry of Health, Columbia University, American College of Nurse Midwives. Evaluation of the safe and clean delivery training program for Health Extension Workers in Ethiopia. Addis Ababa, Ethiopia, 2014.

22. Central Statistical Agency (CSA) [Ethiopia] and ICF. Ethiopia Demographic and Health Survey 2016: key indicators report. Addis Ababa, Ethiopia, and Rockville, Maryland, USA: CSA and ICF 2016.

23. Federal Democratic Republic of Ethiopia Ministry of Health. Health sector development programme IV annual performance report, EFY 2007 (2014/15). Addis Ababa, Ethiopia: Federal Ministry of Health 2015.

24. The Federal Democratic Republic of Ethiopia Ministry of Health. Health Sector Transformation Plan 2015/16 - 2019/20 (2008-2012 EFY). Addis Ababa, Ethiopia: Federal Ministry of Health 2015.

25. Federal Democratic Republic of Ethiopia Ministry of Health. Health sector transformation plan I. Annual performance report, EFY 2008 (2015/16). Addis Ababa, Ethiopia: Federal Ministry of Health 2016.

For peer review only

26. Kok MC, Kea AZ, Datiko DG, et al. A qualitative assessment of health extension workers' relationships with the community and health sector in Ethiopia: opportunities for enhancing maternal health performance. *Hum Resour Health* 2015;13(1):80 doi: 10.1186/s12960-015-0077-4.

27. Wang H, Tesfaye R, Ramana G, et al. Ethiopia health extension program: an institutionalized community approach for universal health coverage. Washington D.C, USA: World Bank 2016.

28. Banteyerga H. Boosting maternal health care seeking behavior in rural low income communities: a case study of West Gojam and South Wollo Zones in Amhara, Ethiopia. *American Journal of Health Research* 2014;2(6):378-86.

29. Godefay H, Kinsman J, Admasu K, et al. Can innovative ambulance transport avert pregnancy-related deaths? One-year operational assessment in Ethiopia. *J Glob Health* 2016;6(1):010410 doi: 10.7189/jogh.06.010410.

30. Atnafu H, Belete Z, Kinfu H, et al. Can a community-based maternal care package in rural Ethiopia increase the use of health facilities for childbirth and reduce the stillbirth rate? *Int J womens Health* 2016;8:421-8 doi: 10.2147/ijwh.s104160.

31. Michie S, van Stralen MM, West R. The behaviour change wheel: a new method for characterising and designing behaviour change interventions. *Implement Sci* 2011;6:42 doi: 10.1186/1748-5908-6-42.

32. Ostebo MT, Cogburn MD, Mandani AS. The silencing of political context in health research in Ethiopia: why it should be a concern. *Health Policy Plan* 2018;33(2):258-70.

33. Bohren MA, Hunter EC, Munthe-Kaas HM, et al. Facilitators and barriers to facility-based delivery in low- and middle-income countries: a qualitative evidence synthesis. *Reprod Health* 2014;11(1):71 doi: 10.1186/1742-4755-11-71.

34. Godlonton S, Okeke EN. Does a ban on informal health providers save lives? Evidence from Malawi. *J dev Econ* 2016;118:112-32 doi: 10.1016/j.jdeveco.2015.09.001.

35. Turinawe EB, Rwemisisi JT, Musinguzi LK, et al. Traditional birth attendants (TBAs) as potential agents in promoting male involvement in maternity preparedness: insights from a rural community in Uganda. *Reprod Health* 2016;13:24 doi: 10.1186/s12978-016-0147-7.

36. Rudrum S. Traditional birth attendants in rural Northern Uganda: policy, practice, and ethics. *Health Care Women Int* 2016;37(2):250-69 doi: 10.1080/07399332.2015.1020539.

37. Sarelin A. Modernisation of maternity care in Malawi. *Nord J Hum Rights* 2014;32(4):331-51 doi: 10.1080/18918131.2014.957463[|.

For peer review only

38. Banda E. Stakeholders' perceptions of the changing role of traditional birth attendants in the rural areas of Central West Zone, Malawi: a mixed methods study. PhD thesis, University of the Witwatersrand, 2013.

39. Rishworth A, Dixon J, Luginaah I, et al. "I was on the way to the hospital but delivered in the bush": maternal health in Ghana's Upper West Region in the context of a traditional birth attendants' ban. *Soc Sci Med* 2016;148:8-17 doi: 10.1016/j.socscimed.2015.11.018.

40. Cheelo C, Nzala S, Zulu JM. Banning traditional birth attendants from conducting deliveries: experiences and effects of the ban in a rural district of Kazungula in Zambia. *BMC Pregnancy Childbirth* 2016;16(1):323 doi: 10.1186/s12884-016-1111-9.

41. Eggermont M. The choice of child delivery is a European human right. *Eur J Health Law* 2012;19(3):257-69.

42. Stromerova Z. Midwifery in East Europe: challenges and solutions. *Midwifery Matters* 2012(133):4-6.

43. Melberg A, Diallo AH, Ruano AL, et al. Reflections on the unintended consequences of the promotion of institutional pregnancy and birth care in Burkina Faso. *PLoS One* 2016;11(6).

44. Hornik RC. Exposure: Theory and evidence about all the ways it matters. *Soc Mark Q* 2002;8(3):31-37 doi: 10.1080/15245000214135.

45. Murray J, Remes P, Ilboudo R, et al. The saturation+ approach to behavior change: case study of a child survival radio campaign in Burkina Faso. *Glob Health Sci Pract* 2015;3(4):544-56 doi: 10.9745/ghsp-d-15-00049.

46. Aubel J, Toure I, Diagne M. Senegalese grandmothers promote improved maternal and child nutrition practices: the guardians of tradition are not averse to change. *Soc Sci Med* 2004;59(5):945-59 doi: 10.1016/j.socscimed.2003.11.044.

47. Dumbaugh M, Tawiah-Agyemang C, Manu A, et al. Perceptions of, attitudes towards and barriers to male involvement in newborn care in rural Ghana, West Africa: a qualitative analysis. *BMC pregnancy and childbirth* 2014;14:269 doi: 10.1186/1471-2393-14-269.

48. Iganus R, Hill Z, Manzi F, et al. Roles and responsibilities in newborn care in four African sites. *Trop Med Int Health* 2015;20(10):1258-64 doi: 10.1111/tmi.12550.

49. Polit DF, Beck CT. Generalization in quantitative and qualitative research: myths and strategies. *Int J Nurs Stud* 2010;47(11):1451-8 doi: 10.1016/j.ijnurstu.2010.06.004.

50. Finfgeld-Connett D. Generalizability and transferability of meta-synthesis research findings. *J Adv Nurs* 2010;66(2):246-54 doi: 10.1111/j.1365-2648.2009.05250.x.

For peer review only - http://bmjopen.bmj.com/site/about/guidelines.xhtml

For peer review only

For peer review only - http://bmjopen.bmj.com/site/about/guidelines.xhtml

Reporting checklist for qualitative study.

Based on the SRQR guidelines.

Instructions to authors

Complete this checklist by entering the page numbers from your manuscript where readers will find each of the items listed below.

Your article may not currently address all the items on the checklist. Please modify your text to include the missing information. If you are certain that an item does not apply, please write "n/a" and provide a short explanation.

Upload your completed checklist as an extra file when you submit to a journal.

In your methods section, say that you used the SRQR reporting guidelines, and cite them as:

O'Brien BC, Harris IB, Beckman TJ, Reed DA, Cook DA. Standards for reporting qualitative research: a synthesis of recommendations. Acad Med. 2014;89(9):1245-1251.

		Reporting Item	Page Number
	#1	Concise description of the nature and topic of the study identifying the study as qualitative or indicating the approach (e.g. ethnography, grounded theory) or data collection methods (e.g. interview, focus group) is recommended	4
	#2	Summary of the key elements of the study using the abstract format of the intended publication; typically includes background, purpose, methods, results and conclusions	2
Problem formulation	#3	Description and signifcance of the problem / phenomenon studied: review of relevant theory and empirical work; problem statement	3
Purpose or research question	#4	Purpose of the study and specific objectives or questions	3
Qualitative approach and research paradigm	#5	Qualitative approach (e.g. ethnography, grounded theory, case study, phenomenolgy, narrative research) and	3

For peer review only

		guiding theory if appropriate; identifying the research paradigm (e.g. postpositivist, constructivist / interpretivist) is also recommended; rationale. The rationale should briefly discuss the justification for choosing that theory, approach, method or technique rather than other options available; the assumptions and limitations implicit in those choices and how those choices influence study conclusions and transferability. As appropriate the rationale for several items might be discussed together.	
Researcher characteristics and reflexivity	#6	Researchers' characteristics that may influence the research, including personal attributes, qualifications / experience, relationship with participants, assumptions and / or presuppositions; potential or actual interaction between researchers' characteristics and the research questions, approach, methods, results and / or transferability	6
Context	#7	Setting / site and salient contextual factors; rationale	4
Sampling strategy	#8	How and why research participants, documents, or events were selected; criteria for deciding when no further sampling was necessary (e.g. sampling saturation); rationale	4
Ethical issues pertaining to human subjects	#9	Documentation of approval by an appropriate ethics review board and participant consent, or explanation for lack thereof; other confidentiality and data security issues	6
Data collection methods	#10	Types of data collected; details of data collection procedures including (as appropriate) start and stop dates of data collection and analysis, iterative process, triangulation of sources / methods, and modification of procedures in response to evolving study findings; rationale	4-6
Data collection instruments and technologies	#11	Description of instruments (e.g. interview guides, questionnaires) and devices (e.g. audio recorders) used for data collection; if / how the instruments(s) changed over the course of the study	5
Units of study	#12	Number and relevant characteristics of participants, documents, or events included in the study; level of	5

For peer review only

		participation (could be reported in results)	
Data processing	#13	Methods for processing data prior to and during analysis, including transcription, data entry, data management and security, verification of data integrity, data coding, and anonymisation / deidentification of excerpts	6
Data analysis	#14	Process by which inferences, themes, etc. were identified and developed, including the researchers involved in data analysis; usually references a specific paradigm or approach; rationale	6
Techniques to enhance trustworthiness	#15	Techniques to enhance trustworthiness and credibility of data analysis (e.g. member checking, audit trail, triangulation); rationale	6
Syntheses and interpretation	#16	Main findings (e.g. interpretations, inferences, and themes); might include development of a theory or model, or integration with prior research or theory	7-10
Links to empirical data	#17	Evidence (e.g. quotes, field notes, text excerpts, photographs) to substantiate analytic findings	7-10
Intergration with prior work, implications, transferability and contribution(s) to the field	#18	Short summary of main findings; explanation of how findings and conclusions connect to, support, elaborate on, or challenge conclusions of earlier scholarship; discussion of scope of application / generalizability; identification of unique contributions(s) to scholarship in a discipline or field	11-12
Limitations	#19	Trustworthiness and limitations of findings	12
Conflicts of interest	#20	Potential sources of influence of perceived influence on study conduct and conclusions; how these were managed	12
Funding	#21	Sources of funding and other support; role of funders in data collection, interpretation and reporting	1

The SRQR checklist is distributed with permission of Wolters Kluwer © 2014 by the Association of American Medical Colleges. This checklist can be completed online using https://www.goodreports.org/, a tool made by the EQUATOR Network in collaboration with Penelope.ai

For peer review only
